# Visualizing active viral infection reveals diverse cell fates in synchronized algal bloom demise

**DOI:** 10.1073/pnas.2021586118

**Published:** 2021-03-11

**Authors:** Flora Vincent, Uri Sheyn, Ziv Porat, Daniella Schatz, Assaf Vardi

**Affiliations:** ^a^Department of Plant and Environmental Sciences, Weizmann Institute of Science, 7610001 Rehovot, Israel;; ^b^Flow Cytometry Unit, Life Sciences Core Facilities, Weizmann Institute of Science, 7610001 Rehovot, Israel

**Keywords:** giant virus, algal blooms, viral life cycle, single cell, smFISH

## Abstract

Marine viruses are the most abundant biological entity in the ocean and are considered as major evolutionary drivers of microbial life [C. A. Suttle, *Nat. Rev. Microbiol.* 5, 801–812 (2007)]. Yet, we lack quantitative approaches to assess their impact on the marine ecosystem. Here, we provide quantification of active viral infection in the bloom forming single-celled phytoplankton *Emiliania huxleyi* infected by the large virus EhV, using high-throughput single-molecule messenger RNA in situ hybridization (smFISH) of both virus and host transcripts. In natural samples, viral infection reached only 25% of the population despite synchronized bloom demise exposing the coexistence of infected and noninfected subpopulations. We prove that photosynthetically active cells chronically release viral particles through nonlytic infection and that viral-induced cell lysis can occur without viral release, thus challenging major assumptions regarding the life cycle of giant viruses. We could also assess active infection in cell aggregates linking viral infection and carbon export to the deep ocean [C. P. Laber *et al.*, *Nat. Microbiol.* 3, 537–547 (2018)] and suggest a potential host defense strategy by enrichment of infected cells in sinking aggregates. Our approach can be applied to diverse marine microbial systems, opening a mechanistic dimension to the study of biotic interactions in the ocean.

## Significance

Despite years of research in aquatic virology, we remain unable to estimate viral-induced mortality in the ocean and, consequently, to resolve viral impact on nutrient fluxes and microbial dynamics. Here, we assess active infection in algal single cells by subcellular visualization of virus and host transcripts, revealing the coexistence of infected and noninfected subpopulations. We revisit major assumptions of a giant virus’ life cycle: cells can produce virions without lysing and can lyse without producing virions. In a natural algal bloom, only 25% of cells were infected, highlighting the importance of other mortality agents. Enrichment of infected cells in cell aggregates suggests potential host defense strategies. Our approach opens a mechanistic dimension to the study of marine microbial interactions.

Microbial communities play a crucial role in shaping marine ecosystems and biogeochemical cycles (1–3). In particular, abundant photosynthetic microeukaryotes and cyanobacteria (phytoplankton) contribute to half of the total primary production on Earth and form the basis of the marine food web (4–6). Phytoplankton blooms can undergo synchronized demise following infection by viruses, present in up to 10^8^ viruses per liter of seawater (3, 7). One of the main challenges in aquatic virology is to quantify how viruses, through control of the host metabolism and abundance, remodel nutrient fluxes within major biogeochemical cycles and impact the microbial community via the viral shunt (8–10). This challenge meets the critical need to assess active viral infection among host cells (virocells) within a complex microbial consortium in the marine environment.

Approaches such as bulk RNA sequencing (RNA-Seq) (11, 12), Digital Droplet PCR (13), polony (14), viral-BONCAT (15), phageFISH (16), and single-cell genomics (17, 18) have considerably expanded the viral ecology toolbox and provided insights into viral diversity and virus-encoded auxiliary metabolic genes that remodel the cell metabolism (19). However, these methods do not report active viral infection and its heterogeneity among individual virocells, which are defined as “the living form of the virus [...] that is, in its intra-cellular form” (ref. 20, p. 233). Recent advances in single-cell transcriptomics have greatly improved our understanding of host–virus interactions by uncovering diverse infection states and dynamics within individual virocells (21, 22). However, these methods remain expensive and restricted to laboratory cultures and lack the ability to visualize infection on the subcellular level that can provide fresh insights into the complex life cycle of viruses and the interplay with their host. Furthermore, we are still missing the ability to quantify active infection at the single-cell level in natural blooms that will enable the definition of diverse infection states and cell fates of the virocells. Single-Molecule mRNA Fluorescent In situ Hybridization (smFISH) enables detection, enumeration, and localization of single messenger RNA (mRNA) molecules within morphologically intact individual cells (23). In this approach, mRNAs are fluorescently labeled whereby probe fluorescence intensity becomes a proxy for gene expression levels (24). Therefore, smFISH represents a tool to visualize transcripts involved in specific host–virus interactions within microbial consortia.

Here, we provide unprecedented quantification of active viral infection and host response of the cosmopolitan unicellular alga *Emiliania huxleyi* both in the laboratory and during algal bloom succession in the natural environment. Demise of *E. huxleyi*’s massive oceanic blooms is attributed to infection by its large double-strand DNA (dsDNA) virus EhV (3, 25, 26), a member of the nucleo-cytoplasmic large DNA viruses (27). We used smFISH to quantify host and virus mRNA and tracked infection dynamics at a single-virocell level by high-throughput imaging flow cytometry. Our study provides unique insights into the life cycle and ecology of an ecologically important giant virus.

## Results

**Fig. 1.**
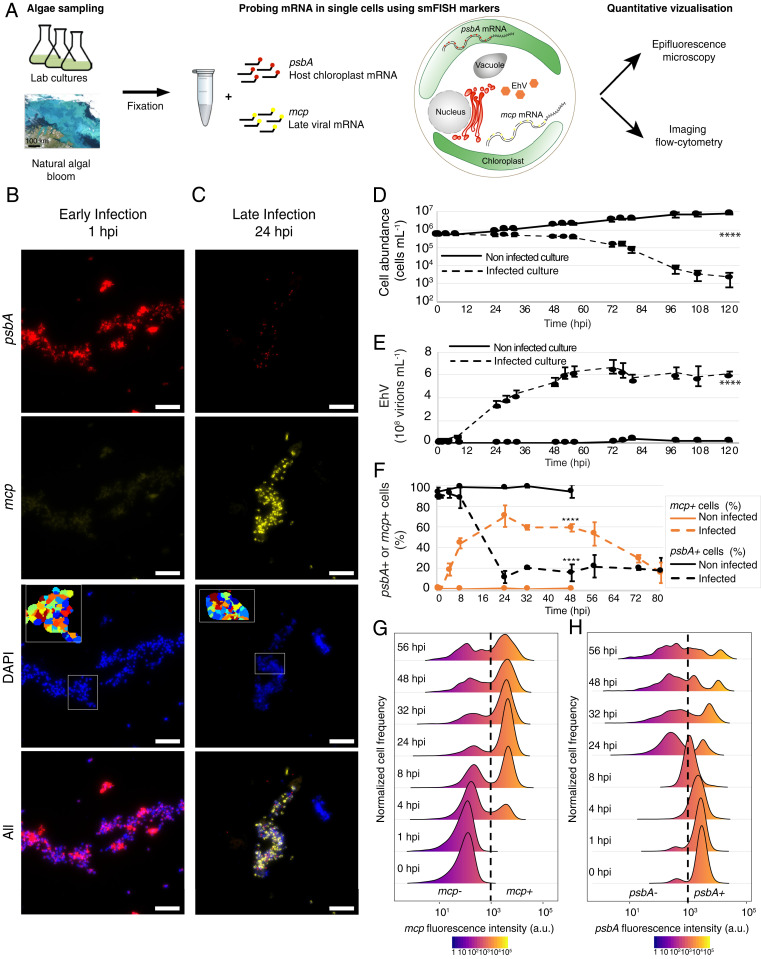
High-throughput visualization of active viral infection in algal virocells using mRNA smFISH. (*A*) Simplified workflow of sample processing and data acquisition. After initial fixation, samples were hybridized to custom-made fluorescent probes targeting the host (*psbA*) and the virus (*mcp*) mRNA and were subjected to epifluorescence microscopy (high resolution) and imaging flow cytometry (high throughput) analyses. (*B* and *C*) Epifluorescence images of an infected culture of *E. huxleyi* cells at 1 and 24 hpi (early and late infection, respectively) in each channel. (Scale bar: 20 μm.) In the DAPI channel, the *Inset* images depict segmented nuclei (see *SI Appendix*, Fig. S12 for a full picture). (*D* and *E*) Cell and virion concentrations, respectively, of infected and noninfected cultures during infection of *E. huxleyi* cells by EhV. Infection was quantified using flow cytometry at a high virus:host ratio. (*F*) Fraction of *mcp*+ and *psbA*+ cells in infected (dashed line) and noninfected (solid line) cultures. Values are presented as the mean ± SD, *n* = 3. ****P < 0.0001 tested with linear mixed model fit by REML. T-tests use Satterthwaite’s method. (*G* and *H*) Distribution of *mcp* and *psbA* fluorescence intensity values in *E. huxleyi* single cells acquired by ImageStreamX during a time course of viral infection. The dashed line depicts the intensity threshold used to define *mcp*+ and *psbA*+ cells according to the fluorescent intensity (10^3^ a.u.).

In order to quantify dynamics of active viral infection, a time course of *E. huxleyi* infection at high virus:host ratio was performed, comparing infected and noninfected *E. huxleyi* cultures. Samples were collected to count cell and viral abundances as well as for smFISH probing. To track the metabolic state of *E. huxleyi* during viral infection, we monitored the mRNA expression of *psbA*, a chloroplast-encoded gene of the D1 protein. D1 is a major component of the photosystem II complex involved in the first step of the light reaction during photosynthesis. D1 has a rapid turnover, requiring constant transcription of *psbA* (28), and is essential for optimal viral infection of *E. huxleyi* (29). Concomitantly, we followed the expression of the viral *mcp* gene that encodes the major capsid protein expressed at the late stage of the viral infection (22) (Fig. 1*A*). Epifluorescence microscopy was used to analyze the samples at 1 and 24 h postinfection (hpi) and showed an increase in the fraction of cells that express *mcp* at 24 hpi compared to 1 hpi, in concert with a profound loss of *psbA* expression, thus validating our approach (Fig. 1 *B* and *C*). Host-cell abundance of infected cultures remained relatively stable at a cell density of 5 × 10^5^ cells per milliliter before decreasing at 48 hpi during the onset of the lytic phase (Fig. 1*D*). The first increase in extracellular concentration of virions occurred at 8 hpi before reaching a plateau of 5.3 × 10^8^ virions per milliliter at 56 hpi (Fig. 1*E*). In order to quantify infection dynamics at a single-cell resolution in a high-throughput manner, smFISH samples were probed for *mcp* and *psbA*, stained with DAPI, and acquired using a multispectral imaging flow cytometer (ImageStreamX). In the infected culture, *mcp*+ cells represented 19% of the population at 4 hpi, 75% at 24 hpi, and plateaued at 60% between 32 and 48 hpi before declining (Fig. 1*F*). Host *psbA* expression showed that 98% of the cells in the noninfected culture remained *psbA*+, in contrast with a sharp drop to 12% in the infected cultures between 8 and 24 hpi (Fig. 1*F*). Infected cells, defined by their *mcp*+ signal, showed large variability in the amounts of *mcp* mRNA per cell, reflected by a wide dynamic range of intensity values of the *mcp* probe per cell (from 10^3^ to 10^5^ arbitrary units [a.u.] of fluorescence), suggesting cell-to-cell heterogeneity in levels of viral infection (Fig. 1*G*). Simultaneously, we detected a bimodal response in the infected culture with high and low *psbA* expression (threshold at 1.1 × 10^3^ a.u. fluorescence) indicating the loss of photosynthetically active cells in a distinct subpopulation (Fig. 1*H*). smFISH therefore enables dual quantification of host and viral genes during infection dynamics across thousands of single cells.

**Fig. 2.**
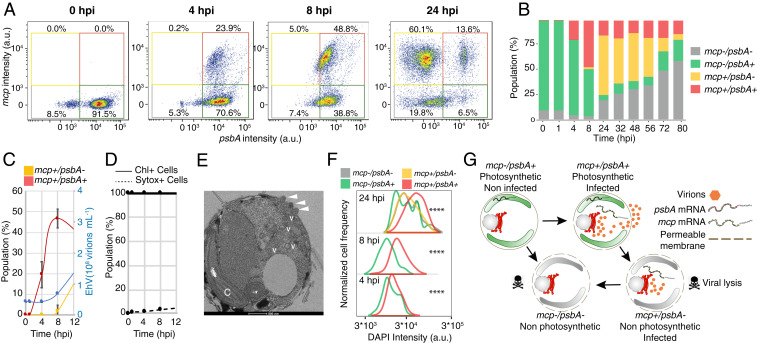
Heterogeneity in transcriptional states during viral infection reveals distinct potential cell fates. (*A*) To investigate the heterogeneity of *E. huxleyi* virocells during infection, we plotted host and virus mRNA coexpression per cell at different time points of hours postinfection. The *x* axis represents the value of the probe intensity (in fluorescent arbitrary units) targeting the *psbA* host gene, and the *y* axis represents the value of the probe intensity targeting the viral *mcp* gene. Using a threshold of ∼10^^3^ a.u. of fluorescence to define both *mcp*+ (*y* axis) and *psbA*+ (*x* axis) cells, we define four subpopulations as a combination of *mcp* and *psbA* signals: *mcp–*/*psbA*+ (green gate), *mcp*+/*psbA*+ (red gate), *mcp*+/*psbA–* (yellow gate), and *mcp–*/*psbA–* (gray gate) (*B*) Relative abundance of the four subpopulations throughout the course of infection. (*C*) Virion production (blue) and *mcp*+ dynamics in the first 12 hpi (*mcp*+/*psbA*+ in red, *mcp*+/*psbA–* in yellow). (*D*) Fraction of cells in the population with high chlorophyll signal (Chl+) and percentage of cells with permeable membranes (Sytox+). Values are presented as the mean ± SD; *n* = 3. (*E*) Transmission electron microscopy of an infected *E. huxleyi* cell at 4 hpi. White arrowheads indicate budding viruses. C: chloroplast; V: immature intracellular viruses. (*F*) Comparison of DNA content based on DAPI intensity between the four subpopulations (with more than 25 events per population) throughout infection. Comparison of DAPI intensity was performed with a linear mixed model fit by REML. T-tests use Satterthwaite’s method. ****P < 0.0001. (*G*) Conceptual scheme of the potential cell fates of different subpopulations.

To investigate *E. huxleyi* virocell heterogeneity in infected cultures, we plotted host and virus mRNA expressions in parallel and revealed four distinct coexisting subpopulations (Fig. 2*A*) that we quantified throughout the course of infection (Fig. 2*B* and *SI Appendix*, Fig. S1*A*). At 0 and 1 hpi, most of the cells were *mcp–*/*psbA*+ (green gate), indicating photosynthetically active cells (Fig. 2*A**, *0 hpi). Cells from noninfected cultures appeared in this gate at all time points. At 4 hpi, 24% of the cells were both *mcp*+ and *psbA*+ (red gate, Fig. 2*A**, *4 hpi), coexpressing viral and host genes. Two new subpopulations were clearly distinguishable at 24 hpi: 60% of the cells were *mcp*+/*psbA–* (yellow gate) as a consequence of *psbA* transcription shutdown or rapid *psbA* mRNA degradation; 20% of the cells were *mcp–*/*psbA–* (gray gate, Fig. 2*A**, *24 hpi). This subpopulation can originate from *mcp–*/*psbA*+ cells experiencing gradual photosynthetic shutdown, illustrated by the large heterogeneity in *psbA* intensity within the *mcp–*/*psbA*+ subpopulation or from *mcp*+/*psbA–* cells that interrupted *mcp* transcription. We further compared these four subpopulations with single-cell RNA-Seq data (22) and show that they corresponded to distinct stages of viral progression, as defined by the temporal succession of viral gene expression (*SI Appendix*, Fig. S2). Intriguingly, viral release increased between 4 and 8 hpi, when infected cells were observed only in the *mcp*+/*psbA*+ subpopulation (Fig. 2*C*). In parallel, algal cell abundance remained stable, and only a small fraction of cells displayed membrane permeability based on *Sytox*+ staining, indicating the absence of cell lysis (Fig. 2*D*). This suggests that photosynthetically active infected cells (*mcp*+/*psbA*+) are responsible for early viral release, indicating a nonlytic infection phase. This was supported by transmission electron microscopy (TEM) of infected cells at 4 hpi, displaying viral egress from undamaged cells that display intact chloroplast morphology and thylakoid stacking (Fig. 2*E*). *mcp*+/*psbA*+ cells had an average DAPI intensity four times higher than other subpopulations and showed strong positive correlation between the *mcp* signal and DAPI intensity (*SI Appendix*, Fig. S3). This is likely a consequence of the virus-induced de novo nucleotide synthesis required to meet the high metabolic demand of giant viruses (30). Utilizing de novo nucleotide synthesis rather than host DNA degradation or nucleotide recycling, which would compromise cell viability, may serve as a viral hijacking strategy at early stages of infection (Fig. 2*F* and *SI Appendix*, Fig. S4*B*). Among *mcp*+/*psbA–* cells at 24 hpi, *mcp* and DAPI signals were colocalized, suggesting overlap between viral genome dsDNA and transcripts of viral structural proteins (*SI Appendix*, Fig. S4*D*). Average *psbA* intensity in the *mcp*+/*psbA*+ subpopulation was higher than in *mcp–*/*psbA*+ cells at 8 and 24 hpi (*SI Appendix*, Fig. S5). This could reflect either enhanced *psbA* transcription in infected cells or down-regulation of *psbA* translation leading to accumulation of mRNA, as shown for some plant viruses (31), although transcription and translation can be decoupled (32). At the population level in the first 12 h, despite identical cell counts between noninfected and infected cultures, mean cell chlorophyll intensity decreased in the infected culture suggesting chlorophyll degradation, coherent with the general loss of photosynthetic transcripts (*SI Appendix*, Fig. S6). Higher *psbA* expression in the *mcp*+ population emphasizes the interdependence between optimality of infection (*mcp* expression) and host photosynthetic regulation (*psbA* expression).

Based on intracellular virocell dynamics, we quantified fundamental parameters in the life cycle of a giant marine virus. We assumed that all cells encounter an infectious viral particle within 30 min after infection as experiments were conducted at high virus:host ratio (*SI Appendix*, Table S1). We further consider the *mcp* mRNA as a strong signal for active viral infection. We estimate that the eclipse phase, the time elapsed between successful cell infection and the initiation of virus production (33), occurs within the first 4 h, based on the induction of *mcp*+/*psbA*+ cells, without an increase in virion concentration (Fig. 2*C*). The onset of the maturation phase, during which viral progeny are released, occurs within 8 h as suggested by previous studies (34). The distinct infection states can be associated with unique cell fates: photosynthetically active infected cells (*mcp*+/*psbA*+) are responsible for early viral release, most likely through budding (34, 35), while *mcp*+/*psbA–* likely contribute less to this process. Virion egress from the host cells without induction of cell death may provide an optimal coexistence mechanism during bloom initiation phase (36). The *mcp–*/*psbA–* subpopulation correlated with Sytox Green staining at later time points (*SI Appendix*, Fig. S1*B*), suggesting that, after 56 hpi, a substantial fraction of the cells contain compromised cell membranes and induce cell death without releasing viruses. These cells can die from accumulation of DNA damage (37), exposure to cytotoxic viral glycosphingolipids (38), or abortive infection. The *mcp–*/*psbA–* and *mcp–*/*psbA*+ cells might serve as the seed for the resistant phenotypes, often observed in a small subpopulation that recovers from viral infection in the so-called “Cheshire cat” escape strategy (39). The transition between different phenotypic states could be mediated by production of infochemicals, such as viral glycosphingolipids (38, 40), or by extracellular vesicles (41) (Fig. 2*G*).

**Fig. 3.**
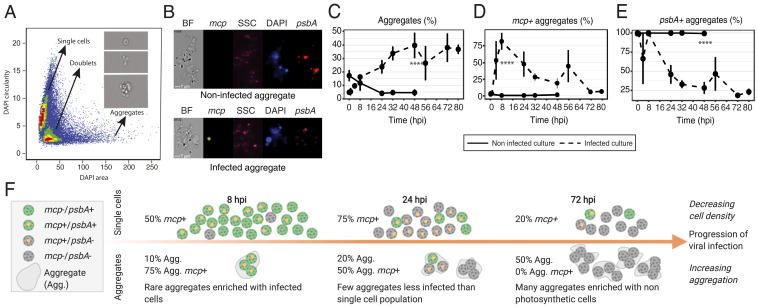
Quantification of viral infection within aggregates formed during infection. (*A*) Based on the DAPI mask area and circularity (the degree of the mask’s deviation from a circle), three populations are identified as single cells, doublets, and aggregates (aggregates are defined by a DAPI area above 60 μm^2^). A representative image of each population is presented in the *Inset*. (*B*) Imaging flow-cytometry images of noninfected (*Top*) and infected (*Bottom*) aggregates in the brightfield (BF), *mcp* (yellow), Side Scatter (SSC as a proxy for calcification, pink), DAPI (blue), and *psbA* (red) channels. (*C*) Fraction of aggregates of all DAPI+ events in infected (dashed line) and noninfected (solid line) cultures. (*D* and *E*) Quantification of *mcp*+ and *psbA*+ aggregates, respectively, throughout the time course of infected (dashed line) and noninfected (solid line) cultures. In *C*–*E*, values are presented as the mean ± SD; *n* = 3; ****P < 0.0001 (*t* test). (*F*) Schematic representation of active infection in single cells versus aggregates throughout progression of viral infection. Illustration produced with Biorender.

The consequences of viral infection in the ocean are not limited to the fate of the individual host cell. Viral infection can also lead to aggregate formation, mediated by production of transparent exopolymer particles (TEP) consisting of acidic polysaccharides, which are classically quantified in bulk by staining with Alcian Blue (42). Aggregates accelerate sinking of phytoplankton biomass, contribute to “marine snow,” and enhance carbon export to the deep sea (3). In order to assess the contribution of viral infection to aggregate formation, we quantified aggregated *E. huxleyi* cells and characterized the infection state of individual cells within the aggregates using the smFISH approach (Fig. 3 *A* and *B*). Following infection, up to 40% of the algal cells (DAPI-positive fraction) were found within aggregates (Fig. 3*C*). At the early stage of infection, less than 10% of the cells are found in aggregates, mostly infected (75% of the aggregates contained at least one infected cell thereafter named *mcp*+) and photosynthetically active (100% were *psbA*+) (Fig. 3 *D* and *E*). At the onset of the lytic phase (48 hpi), aggregates were highly abundant, less infected (25% were *mcp*+), and less photosynthetically active (30% were *psbA*+). In infected cultures at 8 hpi, the fraction of *mcp*+ aggregates was higher than the fraction of *mcp*+ single cells (75 and 50%, respectively, Figs. 1*F* and 3*D*). We hypothesize that aggregates enriched with infected cells trap newly produced virions and thus can export them out of the photic zone and prevent further dissemination of viruses in the bloom, suggesting a host defense strategy (3, 26) (Fig. 3*F*). The overall presence of infected cells in aggregates corroborates previous observations of TEP-attached viruses (42, 43). This could further explain the estimates of 10^9^ viruses per gram of marine sediment, some being infectious, that may serve as a possible inoculum to infect subsequent blooms (44, 45). The asymmetry of infection levels between single cells and aggregates is reflected at a later time point, when aggregates become abundant but show no evidence of active infection, despite 20% of infected cells in the single-cell population.

**Fig. 4.**
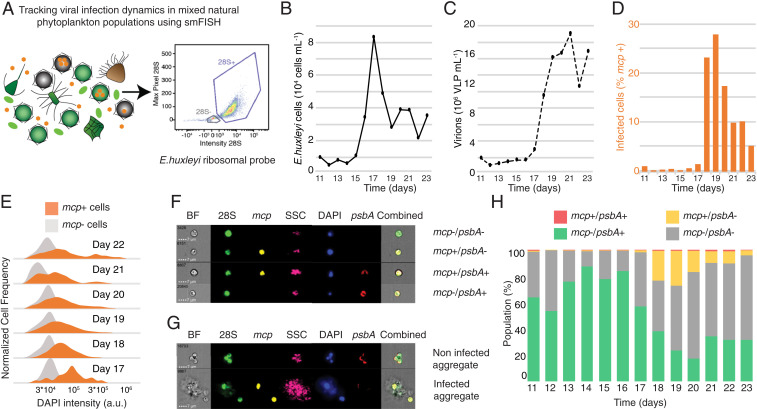
Visualization of active viral infection during a natural *E. huxleyi* bloom. (*A*) Applying smFISH to track *E. huxleyi* virocells within mixed natural microbial populations. A probe specifically targeting the 28S ribosomal RNA of *E. huxleyi* was used to identify our host of interest, based on the intensity and maximum pixel of the probe signal in flow-cytometry plots, along with the *mcp* and *psbA* probes. (*B* and *C*) Abundance of calcified *E. huxleyi* cells and EhV-like particles (VLP), respectively, measured by flow cytometry throughout bloom succession in a mesocosm experiment. (*D*) Fraction of infected (*mcp*+) *E. huxleyi* virocells, measured by smFISH analysis throughout bloom succession. (*E*) Comparison of DAPI intensities between infected and noninfected *E. huxleyi* populations throughout bloom succession. (*F* and *G*) Visualizing subpopulations of *E. huxleyi* single cells and aggregates, respectively, using ImageStreamX (SSC: side scatter). (*H*) Quantification of the relative abundance of the four subpopulations in single cells throughout the bloom succession by coprobing of host and viral mRNA (*psbA* and *mcp*, respectively).

One of the fundamental knowledge gaps in aquatic virology stems from the challenge to quantify active viral infection in natural populations and mechanistically resolve the viral impact on microbial ecosystems. We therefore conducted a mesocosm experiment in a fjord near Bergen, Norway, and collected samples for smFISH analysis across different phases of an *E. huxleyi* bloom. We quantified viral infection specifically in *E. huxleyi* host cells by using a 28S ribosomal probe unique to that alga, a *psbA* probe as a proxy for host photosynthetic state, and a probe targeting viral *mcp* transcripts (Fig. 4*A*). smFISH analysis conducted by imaging flow cytometry successfully revealed the fraction of infected *E. huxleyi* virocells during bloom succession, based on over 5,000 cells at each time point (*SI Appendix*, Fig. S7). From the initiation of the bloom to the peak of *E. huxleyi* abundance on day 17 (Fig. 4*B*), only a slight viral increase was observed (Fig. 4*C*), and infection level was minimal (<1% of *mcp*+ cells) (Fig. 4*D*). We detected a tipping point in which the fraction of infected cells rose from 1.51% on day 17 to 23.22% on day 18, while cell abundance declined at the onset of bloom demise. *mcp*+ cells displayed higher DAPI intensity than *mcp–* cells (Fig. 4*E*), confirming induction of de novo nucleotide synthesis in infected cells. Strikingly, the maximal fraction of infected cells did not reach beyond 27% of the *E. huxleyi* population, even at the demise phase of the bloom. Quantification of *mcp* and *psbA* over the course of the bloom (*SI Appendix*, Fig. S8) revealed a major drop in *mcp–*/*psbA*+ cells between days 16 and 18, accompanied by an increase in *mcp*+/*psbA–* cells. From day 19 onward, a large fraction of the *E. huxleyi* population was *mcp–*/*psbA–*, resembling the fraction of cell death during bloom demise (Fig. 4*H*). This population could represent noninfected bystander cells undergoing programmed cell death that is mediated by release of infochemicals (38, 40) or extracellular vesicles (41) from the infected cells. These results suggest that algal bloom demise can be synchronized, despite coexistence of diverse virocell states and only a small subpopulation of infected cells. We suggest that other mortality agents might act in concert with viral lysis to control synchronized bloom demise at a later stage. These top-down regulators may include pathogenic bacteria (46), eukaryotic parasites, and grazers (3, 47, 48). Alternatively, highly synchronized viral infection and release might have occurred in a large fraction of the *E. huxleyi* population in a narrow time frame between days 17 and 18. Indeed, recent reports demonstrate that viruses are intrinsically synchronized with the daily rhythms of their photosynthetic host both in controlled laboratory experiments and in natural populations (49, 50). Additionally, we probed a late viral gene and potentially might detect a higher fraction of infected cells by using an early viral gene probe. Furthermore, the synchronized bloom demise can be explained by increasing cell aggregation at later infection stages (Fig. 3*F*).

## Discussion

Our approach opens up avenues to study specific host*–*virus interactions among a complex microbial consortium in the marine environment. It combines high-throughput morphological and single-molecule transcriptional data, thus providing quantitative subcellular localization of ribosomal and messenger RNA, of both hosts and viruses simultaneously, at single-virocell resolution. Our approach further enables detection of aggregates and small subpopulations, connects intra- and extracellular dynamics, and can be adapted to target viruses and virocells in mixed natural populations by designing probes based on the wealth of available environmental genomics data.

In this study, we unravel fundamental aspects of the life cycle of a large virus by linking scales between single-cell and population-level dynamics, showing viral increase without cell lysis in the *mcp*+/*psbA*+ subpopulation, and cell lysis without viral increase in the *mcp–*/*psbA–* subpopulation. The high-resolution examination of host and virus transcriptional states revealed cell-to-cell heterogeneity in viral infection, mediated by the dependence on the host photosynthetic state, despite synchronized bloom demise. Indeed, viral replication may require photosynthetic activity in order to energize the redirection of carbon flux from the Calvin cycle to the pentose phosphate pathway. This will increase production of NADPH required for de novo DNA synthesis (51) to meet the high demand for nucleotides by the giant viruses and enhance the antioxidant capacity under oxidative stress (52). Thus, a functional convergence arises among viruses infecting phototrophs from diverse evolutionary origin, whereby cyanophages (53), small RNA plant viruses (54), and large algal dsDNA viruses share the ability to manipulate the metabolism of their photosynthetic host. Quantifying the virocell states, through their diverse phenotypes, will help integrate viral-dependent processes into ecosystem models by providing necessary parameters of infection dynamics, such as the fraction of actively infected cells, resistant cells, and the nature of sinking biomass (55). Finally, this quantitative smFISH approach can be expanded to diverse host-pathogen and host-symbiont systems with important ecological significance, adding a mechanistic dimension to the field of microbial ecology.

## Materials and Methods

### Culture Growth and Viral Infection.

The noncalcifying *E. huxleyi* strain CCMP 2090 was used for this study. Cells were cultured in K/2 medium with antibiotics (ampicillin and kanamycin) and incubated at 18 °C with a 16:8 h light–dark illumination cycle. A light intensity of 100 μmol photons m^−2^⋅s^−1^ was provided by cool-white light-emitting diode lights. All experiments started with exponential phase cultures (5 × 10^5^ cells mL^−1^). The virus used for this study is EhV201 propagated on CCMP2090 with antibiotics. Five days before infection, most probable number (MPN) assays were performed to assess the fraction of infectious viruses in the stock (48). *E. huxleyi* was infected with 5:1 multiplicity of infection (MOI) ratio of infectious virus per cell, thereby guaranteeing that all cells encountered an infectious particle 30 min postinfection. The time courses of infected and noninfected cultures were sampled simultaneously in triplicates. A second MPN experiment was performed on the day of the experiment with the same virus and same algae to assess the exact MOI at the beginning of the experiment (*SI Appendix*, Table S1).

### Enumeration of Algal Cell Abundance, Cell Death, and Viral Abundance.

Cells were monitored and quantified using an Eclipse (iCyt) flow cytometer. Cells were identified by plotting the chlorophyll fluorescence (excitation [ex]: 488 nm and emission [em]: 663 to 737 nm) versus side scatter. For extracellular viral counts, samples were stained with SYBR gold (Invitrogen) that was diluted 1:10,000 in Tris–ethylenediaminetetraacetic acid buffer, incubated for 20 min at 80 °C, and cooled to room temperature (RT). Samples were analyzed by an Eclipse flow cytometer (ex: 488 nm; em: 500 to 550 nm), and a minimum of 50,000 events was collected. To analyze cell permeability, samples were stained with a final concentration of 1 μM Sytox Green (Invitrogen), incubated in the dark for 1 h at RT, and analyzed by an Eclipse flow cytometer (ex: 488 nm; em: 500 to 550 nm). An unstained sample was used as a control to eliminate the background signal. The data were exported and analyzed in R.

### *mcp* and *psbA* Probe Design and Conjugation for smFISH.

The smFISH technique identifies a single mRNA based on the binding of multiple small probes targeted to different locations to the mRNA of interest. Fasta sequences of the target genes *mcp* and *psbA* were first submitted to Stellaris Probe Designer to obtain potential probes. We designed 48 probes per gene with a probe length of 20 nucleotides. Probes with more than 70% guanine–cytosine content were discarded. To discard off-targets and decrease nonspecific signal, each probe sequence was blasted against the *E. huxleyi* transcriptome and EhV201 genome. Probes with an off-target gene matching over 17 nucleotides were discarded. A minimum of 20 probes per gene is necessary to detect a single molecule. Validated probes were ordered with 3′ amine groups through the Custom Oligo Service of BioSearch Technologies (Probe_Sequences.xlsx). Fluorophores with succinimidyl ester group were ordered from Click Chemistry Tools: Tetramethylrhodamine (TMR) and sulfo-Cyanine5 (Cy5). Probes were coupled to fluorophores and purified as described in ref. 23. All the conjugated probes of a given gene were used as a mixture each time that we targeted the expression of that gene. Therefore, the *mcp* mRNA was detected using 47 probes of 20 nucleotides each, and the *psbA* mRNA was detected using 48 probes of 20 nucleotides each. The position of the probes on the reference gene are shown in *SI Appendix*, Fig. S9 *A* and *B*, and their respective positions and sequences are indicated in Dataset S1.

### Sample Fixation and Hybridization for smFISH in Laboratory Samples.

At each time point, 50 mL of each flask was fixed in a cold 1% paraformaldehyde final concentration and incubated for 1 h at 4 °C with gentle agitation. Each sample was then centrifuged for 2 min at 4 °C and 3,000 × *g*. The supernatant was discarded, the pellet resuspended in 1 mL of cryopreservant solution (prepared in 1× phosphate-buffered saline containing 4% paraformaldehyde and 30% sucrose), transferred to a 1.7 mL Eppendorf tube, and incubated for 1 h at 4 °C with agitation. Tubes were then centrifuged for 2 min at 4 °C and 3,000 × *g*, the supernatant was removed and stored at 80 °C until hybridization.

A day before ImageStreamX acquisition, selected samples were thawed and chlorophyll was extracted by a first wash using 900 μL of 70% ethanol applied for 3 min, followed by centrifugation for 3 min at 3,000 × *g* and removal of the supernatant. A second wash was performed with 900 μL of 100% ethanol applied for 3 min, followed by centrifugation for 3 min at 3,000 × *g* and removal of the supernatant. Samples were treated with 500 μL Proteinase K at 10 μg/mL final concentration (Ambion #AM2546) for 10 min at room temperature and washed with 3 min at 3,000 × *g* centrifugation. Samples were then resuspended in 50 μL of hybridization buffer (17.5% formamide concentration) containing equal concentrations of the different target genes at a 0.1 ng/mL final concentration. *Mcp* probes were conjugated with TMR, and *psbA* probes were coupled to Cy5. Hybridization was performed overnight in 30 °C shakers.

The day of ImageStreamX acquisition, hybridization buffer was washed away by centrifugation for 3 min at 3,000 × *g*. Samples were stained for dsDNA with 500 μL of GLOX buffer (prepared in nuclease-free water with 0.4% final concentration of glucose, 2× final concentration sodium chloride–sodium citrate (SSC) Ambion #AM9765, and 10 mM final concentration of Tris, pH 8.0) with DAPI in 10 μg/mL final concentration (except the single stains). DAPI staining was done for 30 min in 30 °C, followed by centrifugation for 3 min at 3,000 × *g*, removal of supernatant, and resuspension in 40 μL of GLOX buffer before being acquired in the ImageStreamX. DAPI was used for several reasons. The first one is technical: DAPI is required for the fluorescent microscope to localize cells when the brightfield channel is not available. We used DAPI to draw the contour of the nucleus using Cell Profiler in Fig. 1 and *SI Appendix*, Fig. S12. DAPI is also important for gating of the cells in the imaging flow cytometer. Gating on brightfield signal and on the DAPI achieves better detection of the cells and thus better downstream statistics. The second major reason to use DAPI is that it provides important information regarding the general DNA content of the cells, its cell-cycle phase, and is a nucleus marker that serves as a reference point in our cells.

The following day, samples were resuspended in 300 μL of GLOX with the 3,3′-dihexyloxacarbocyanine iodide membrane stain at final concentration of 1 μM (ThermoFisher, catalog number D273). Excessive dye was removed by centrifugation for 3 min at 3,000 × *g* and removal of supernatant, followed by resuspension in 4 μL GLOX. A total of 2 μL was deposited on a microscope slide with 2 μL of antibleach solution, and the sample was imaged with the epifluorescent microscope.

### Imaging Flow Cytometry Acquisition, Compensation, and Analysis for Laboratory Samples.

Samples were acquired using the ImageStreamX MarkII machine (ISX, Amnis, Luminex). Three excitation wavelengths were used: 405 nm (DAPI: channel 7 to 50 mW), 561 nm (TMR: channel 3 to 200 mW), and 642 nm (Cy5: channel 11 to 120 mW). For each sample, at least 50,000 cells were acquired and imaged with 60× magnification. Single stained samples were acquired using the compensation wizard of the ImagestreamX. On average, less than 10 μL of 2 × 10^6^ cell stock concentration was necessary to collect the optimal amount of cells, except toward the end of the experiment where cells were scarce.

Data were analyzed using IDEAS6.2 (Amnis, Luminex). The compensation matrix was built using the IDEAS wizard and manually checked before being applied to all the acquired files. Based on the area (the number of microns squared in a mask) and circularity (the degree of the mask’s deviation from a circle) of DAPI, three populations were identified as single cells (mainly, DAPI area <60 a.u.), doublets, and aggregates (mainly, DAPI area >60 a.u.). Single cells were additionally selected in the same focal plane using the BF gradient and contrast (both gradient and contrast measure the sharpness quality of an image by detecting large changes of pixel values in the image). All gates were defined on a single file before being applied to the total data set (*SI Appendix*, Fig. S10). Each file was then manually inspected to check the accuracy of single-cell and aggregate gating. All the data (fluorescent intensities, morphological features, populations) were then exported for each cell of each file for analysis in R. ImagestreamX source data are available on Dryad (https://doi.org/10.5061/dryad.h44j0zpjc) (56). Compensation matrixes are available in .cif files, and analysis of each time point in .daf files.

Similarity was calculated in the IDEAs software. As defined in the IDEAs User Manual (ref. 57, p. 215), “The Similarity feature is the log-transformed Pearson’s correlation coefficient and is a measure of the degree to which two images are linearly correlated within a masked region.” In *SI Appendix*, Fig. S4*D*, we correlate DAPI and *mcp* masks for each cell of two subpopulations (*mcp*+/*psbA*+, *mcp*+/*psbA–*) and plot its distribution. The threshold used to declare colocalization was similarity >1.2.

### RNase Treatment.

*E. huxleyi* cultures were infected with EhV201 at low MOI and fixed in 1% paraformaldehyde for smFISH 24 hpi. Two samples were used to compare *mcp* signal with and without RNase treatment. The sample with RNase treatment was treated with RNase before the Proteinase K treatment, with RNase A from Thermo Fisher (catalog no. EN0531) at a final concentration of 10 μg/mL in a reaction volume of 200 μL of infected *E. huxleyi* culture for 20 min at 37°. Both samples were then processed as usual for *mcp* staining and acquired in the ImageStreamX. Application of RNase treatment on infected samples shows heavily reduced *mcp* signal, confirming that the probes bind to RNA and not to DNA (*SI Appendix*, Fig. S11)

### Epifluorescence Microscope Acquisition and Analysis.

Slides were acquired using a Nikon inverted fluorescence microscope Eclipse Ti2 Series and imaged with a Ixon Ultra 888 camera with 100× magnification, using the Nikon NIS Element Advanced Research Software. Illumination time for each excitation was adjusted at the beginning of each batch acquisition and not modified after that. Each image is composed of 15 0.3-μm stacks. Images were exported in .nd2 format and inspected in Fiji 2 (58). Stacking projection was performed for each file (Image > Stacks > Z-Project > MaxIntensity) on a maximum of 10 stacks. Mean intensity in the background was measured for each channel (Analyze > Measure) and subtracted (Process > Math > Subtract).

To estimate the fraction of infected cells, we used CellProfiler (59) using the PercentPositive pipeline separately for each probe after a maximum projection and background subtraction for each channel separately (*SI Appendix*, Fig. S12).

### TEM.

*E. huxleyi* CCMP2090 was infected with 1:50 volumetric ratio of viral lysate to culture a MOI of 1:1 viral particles per cell. At 4 hpi, 500 mL of culture was collected (8,000 × *g*, 10 min, 20 °C), resuspended in fixation media (2% glutaraldehyde, 4% paraformaldehyde, 2% acrolein in artificial sea water [ASW]), and fixed for at least 24 h at 4 °C. The cells were then washed in ASW and post fixed in 2% osmium tetroxide, 0.5% potassium dichromate, and 0.5% potassium hexacyanoferrate in ASW for 1 h at room temperature, washed again, and stained in bloc with 2% aqueous uranyl acetate for 1 h followed by ethanol dehydration. Samples were infiltrated with increasing concentrations of Epon EMBED 812 (EMS) and polymerized at 60 °C. Thin sections (∼70 nm) obtained with an Ultracut UCT microtome (Leica Microsystems) were poststained with 2% uranyl acetate and Reynold’s lead citrate and examined using an FEI Tecnai T12 TEM operating at 120 kV. Images were recorded on an FEI Eagle 2Kx2K CCD camera.

### Single-Cell Transcriptomics Comparison.

Based on single-cell dual transcriptomics during *E. huxleyi* infection by EhV201 (22), we plotted gene expression of *psbA* (*SI Appendix*, Fig. S13*A*) and *mcp* (*SI Appendix*, Fig. S13*B*) in available cells (*n* = 384) across different time points postinfection. For each gene, we plotted the abundance of the gene’s mRNA molecules per cell (*Left*), followed by the relative expression of that gene with respect to the total number of mRNA molecules in the cell (*Center*), and, finally, the percentage of cells expressing that specific gene (*Right*). Furthermore, we were able to map the single-cell transcriptomics infection states (metacell) into each of the smFISH transcriptional states that were based on *psbA* versus *mcp* coexpression on a single-cell resolution (*SI Appendix*, Fig. S2). The MetaCell method uses the k-nearest neighbor graph partitions (60) for organizing single cells into cohesive groups of cells (metacells) with similar viral gene expression profiles and is defined in our system in ref. 22.

### Mesocosm Experimental Setup.

The mesocosm experiment—Aquacosm-viral-induced microbial succession (Aquacosm-VIMS)—was carried out over 24 d (May 24 to June 17, 2018) in Raunefjorden at the University of Bergen’s Marine Biological Station at Espegrend, Norway (60.27° N; 5.22° E). The experiment consisted of seven enclosure bags made of transparent polyethylene (11 m^3^, 4 m deep, and 2 m wide; 90% photosynthetically active radiation) mounted on floating frames and moored to a raft in the middle of the bay. Each bag was filled with surrounding fjord water and supplemented with nutrients at days 0 to 7 and 13 to 17 at a nitrogen:phosphorous ratio of 16:1 (1.6 μM NaNO_3_, 0.1 μM KH_2_PO_4_). On days 6, 7, and 13 only NaNO_3_ was added. The water in each bag was continuously mixed by pumping air to the bottom of each bag. Samples for flow cytometric counts were taken twice a day, morning (7 AM) and evening (8 to 9 PM), using 50 mL centrifugal tubes and following filtration using a 40 μm cell strainer (61). Calcified *E. huxleyi* were identified using the Eclipse flow cytometer based on high side scattering and high chlorophyll content (*SI Appendix*, Fig. S14). All bags converged to a similar pattern of an *E. huxleyi* bloom and demise, and the current study focuses on bag 4.

### smFISH Specificities for Field Samples.

Sample fixation, storage, and initial chlorophyll washes of field samples were identical to laboratory samples. In order to identify *E. huxleyi* cells, we designed a single probe specific to the 28S region of *E. huxleyi* (62) (EG28‐03, 5′‐TAA​AGC​CCC​GCT​CCC​GGG​TT‐3′, bound to C3-Fluorescein (ex/em = 490/525 nm). Two helper probes were used (Helper A, 5′-GCC​AGG​ACG​GGA​GCT​GGC​CG-3′ and Helper B, 5′-GAG​GCG​CGG​CGC​CGA​GGC​GC-3′) to facilitate unfolding of the 28S rRNA molecule by binding to the flanking regions of the probe of interest EG28; 28S, HelperA, HelperB were used at a final concentration of 0.5 μM at the same time as the *mcp* and *psbA* probes at 0.1 ng/mL final concentration. We further checked the efficiency of our *mcp* probes to detect the most abundant EhV isolated in the mesocosm, EhVM1. Specifically, of the 47 *mcp* probes designed to target the laboratory viral strain EhV201, 34 probes had a 100% match to EhVM1, which is sufficient to obtain high fluorescent signal. The positions of the *mcp* probes on the *mcp* gene of EhVM1 are shown in *SI Appendix*, Fig. S9*C*. EhV201 and EhVM1 shared above 96% identity over the 1,491-bp alignment, suggesting that our probes are efficient in capturing the most abundant EhV of our natural samples. In order to test whether or not our EhV *mcp* probes could bind against the *mcp* sequence of another phycodnaviridae large virus infecting microeukaryotic algae, we mapped the probes against the *mcp* gene of the *Ostreococcus tauri* giant virus OtV (*SI Appendix*, Fig. S9*D*). Only four probes were mapped, with less than 17 nucleotides identity. All samples were probed in 50 μL of 40% formamide hybridization buffer, incubated at 37° overnight. Samples were acquired using the ImageStreamX MarkII machine (ISX, Amnis, Luminex). Four excitation wavelengths were used: 405 nm for the DAPI (DAPI: channel 7 to 50 mW), 488 nm for 28S rRNA (AF488: channel 2 to 200 mW), 561 nm for *mcp* (TMR: channel 3 to 200 mW), and 642 nm for *psbA* (Cy5: channel 11 to 120 mW).

## Supplementary Material

Supplementary File

Supplementary File

## Data Availability

All ImagestreamX data are available on Dryad (https://doi.org/10.5061/dryad.h44j0zpjc).

## References

[1] F. Azam, Microbial control of oceanic carbon flux: The plot thickens. Science 280, 694–696 (1998).

[2] C. A. Suttle, Marine viruses: Major players in the global ecosystem. Nat. Rev. Microbiol. 5, 801–812 (2007). 17853907 10.1038/nrmicro1750

[3] C. P. Laber., Coccolithovirus facilitation of carbon export in the North Atlantic. Nat. Microbiol. 3, 537–547 (2018). 29531367 10.1038/s41564-018-0128-4

[4] T. Fenchel, Marine plankton food chains introduction: The classical view of plankton food chains. Annu. Rev. Ecol. Syst. 19, 19–38 (1988).

[5] C. B. Field, M. J. Behrenfeld, J. T. Randerson, P. Falkowski, Primary production of the biosphere: Integrating terrestrial and oceanic components. Science 281, 237–240 (1998). 9657713 10.1126/science.281.5374.237

[6] Y. M. Bar-On, R. Phillips, R. Milo, The biomass distribution on Earth. Proc. Natl. Acad. Sci. U.S.A. 115, 6506–6511 (2018). 29784790 10.1073/pnas.1711842115 PMC6016768

[7] Y. Lehahn., Decoupling physical from biological processes to assess the impact of viruses on a mesoscale algal bloom. Curr. Biol. 24, 2041–2046 (2014). 25155511 10.1016/j.cub.2014.07.046

[8] S. W. Wilhelm, C. A. Suttle, Viruses and nutrient cycles in the sea. Bioscience 49, 781–788 (1999).

[9] J. S. Weitz, S. W. Wilhelm, Ocean viruses and their effects on microbial communities and biogeochemical cycles. F1000 Biol. Rep. 4, 17 (2012). 22991582 10.3410/B4-17 PMC3434959

[10] J. R. Brum., Patterns and ecological drivers of ocean viral communities. Science 348, 1261498 (2015). 25999515 10.1126/science.1261498

[11] E. T. Sieradzki, J. C. Ignacio-Espinoza, D. M. Needham, E. B. Fichot, J. A. Fuhrman, Dynamic marine viral infections and major contribution to photosynthetic processes shown by spatiotemporal picoplankton metatranscriptomes. Nat. Commun. 10, 1169 (2019). 30862830 10.1038/s41467-019-09106-z PMC6414667

[12] M. Moniruzzaman., Virus-host relationships of marine single-celled eukaryotes resolved from metatranscriptomics. Nat. Commun. 8, 16054 (2017). 28656958 10.1038/ncomms16054 PMC5493757

[13] A. D. Tadmor, E. A. Ottesen, J. R. Leadbetter, R. Phillips, Probing individual environmental bacteria for viruses by using microfluidic digital PCR. Science 333, 58–62 (2011). 21719670 10.1126/science.1200758 PMC3261838

[14] N. Baran, S. Goldin, I. Maidanik, D. Lindell, Quantification of diverse virus populations in the environment using the polony method. Nat. Microbiol. 3, 62–72 (2018). 29085077 10.1038/s41564-017-0045-y PMC5739286

[15] A. L. Pasulka., Interrogating marine virus-host interactions and elemental transfer with BONCAT and nanoSIMS-based methods. Environ. Microbiol. 20, 671–692 (2018). 29159966 10.1111/1462-2920.13996

[16] E. Allers., Single-cell and population level viral infection dynamics revealed by phageFISH, a method to visualize intracellular and free viruses. Environ. Microbiol. 15, 2306–2318 (2013). 23489642 10.1111/1462-2920.12100 PMC3884771

[17] J. M. Labonté., Single-cell genomics-based analysis of virus-host interactions in marine surface bacterioplankton. ISME J. 9, 2386–2399 (2015). 25848873 10.1038/ismej.2015.48 PMC4611503

[18] D. M. Needham., A distinct lineage of giant viruses brings a rhodopsin photosystem to unicellular marine predators. Proc. Natl. Acad. Sci. U.S.A. 116, 20574–20583 (2019). 31548428 10.1073/pnas.1907517116 PMC6789865

[19] B. L. Hurwitz, S. J. Hallam, M. B. Sullivan, Metabolic reprogramming by viruses in the sunlit and dark ocean. Genome Biol. 14, R123 (2013). 24200126 10.1186/gb-2013-14-11-r123 PMC4053976

[20] P. Forterre, The virocell concept and environmental microbiology. ISME J. 7, 233–236 (2013). 23038175 10.1038/ismej.2012.110 PMC3554396

[21] S. Rosenwasser., Unmasking cellular response of a bloom-forming alga to viral infection by resolving expression profiles at a single-cell level. PLoS Pathog. 15, e1007708 (2019). 31017983 10.1371/journal.ppat.1007708 PMC6502432

[22] C. Ku., A single-cell view on alga-virus interactions reveals sequential transcriptional programs and infection states. Sci. Adv. 6, eaba4137 (2020). 32490206 10.1126/sciadv.aba4137 PMC7239649

[23] A. Raj, P. van den Bogaard, S. A. Rifkin, A. van Oudenaarden, S. Tyagi, Imaging individual mRNA molecules using multiple singly labeled probes. Nat. Methods 5, 877–879 (2008). 18806792 10.1038/nmeth.1253 PMC3126653

[24] S. O. Skinner, L. A. Sepúlveda, H. Xu, I. Golding, Measuring mRNA copy number in individual Escherichia coli cells using single-molecule fluorescent in situ hybridization. Nat. Protoc. 8, 1100–1113 (2013). 23680982 10.1038/nprot.2013.066 PMC4029592

[25] W. H. Wilson., Isolation of viruses responsible for the demise of an Emiliania huxleyi bloom in the English Channel. J. Mar. Biol. Assoc. 82, 369–377 (2002).

[26] U. Sheyn., Expression profiling of host and virus during a coccolithophore bloom provides insights into the role of viral infection in promoting carbon export. ISME J. 12, 704–713 (2018). 29335637 10.1038/s41396-017-0004-x PMC5864229

[27] E. V. Koonin, N. Yutin, Origin and evolution of eukaryotic large nucleo-cytoplasmic DNA viruses. Intervirology 53, 284–292 (2010). 20551680 10.1159/000312913 PMC2895762

[28] A. K. Mattoo, J. B. Marder, M. Edelman, Dynamics of the photosystem II reaction center. Cell 56, 241–246 (1989). 2643478 10.1016/0092-8674(89)90897-0

[29] K. Thamatrakoln., Light regulation of coccolithophore host-virus interactions. New Phytol. 221, 1289–1302 (2019). 30368816 10.1111/nph.15459

[30] S. Rosenwasser, C. Ziv, S. G. V. Creveld, A. Vardi, Virocell metabolism: Metabolic innovations during host-virus interactions in the ocean. Trends Microbiol. 24, 821–832 (2016). 27395772 10.1016/j.tim.2016.06.006

[31] K. Lehto, M. Tikkanen, J. B. Hiriart, V. Paakkarinen, E. M. Aro, Depletion of the photosystem II core complex in mature tobacco leaves infected by the flavum strain of tobacco mosaic virus. Mol. Plant Microbe Interact. 16, 1135–1144 (2003). 14651347 10.1094/MPMI.2003.16.12.1135

[32] Y. Taniguchi., Quantifying E. coli proteome and transcriptome with single-molecule sensitivity in single cells. Science 329, 533–538 (2010). 20671182 10.1126/science.1188308 PMC2922915

[33] S. J. Flint, V. R. Racaniello, L. W. Enquist, A. M. Skalka, Principles of Virology: Molecular Biology (Wiley, 2015).

[34] L. C. M. Mackinder., A unicellular algal virus, Emiliania huxleyi virus 86, exploits an animal-like infection strategy. J. Gen. Virol. 90, 2306–2316 (2009). 19474246 10.1099/vir.0.011635-0

[35] B. Knowles., Temperate infection in a virus-host system previously known for virulent dynamics. Nat. Commun. 11, 4626 (2020). 32934228 10.1038/s41467-020-18078-4 PMC7493887

[36] S. Yau., Virus-host coexistence in phytoplankton through the genomic lens. Sci. Adv. 6, eaay2587 (2020). 32270031 10.1126/sciadv.aay2587 PMC7112755

[37] K. D. Bidle, L. Haramaty, J. Barcelos E Ramos, P. Falkowski, Viral activation and recruitment of metacaspases in the unicellular coccolithophore, Emiliania huxleyi. Proc. Natl. Acad. Sci. U.S.A. 104, 6049–6054 (2007). 17392426 10.1073/pnas.0701240104 PMC1838821

[38] A. Vardi., Viral glycosphingolipids induce lytic infection and cell death in marine phytoplankton. Science 326, 861–865 (2009). 19892986 10.1126/science.1177322

[39] M. Frada, I. Probert, M. J. Allen, W. H. Wilson, C. de Vargas, The “Cheshire Cat” escape strategy of the coccolithophore Emiliania huxleyi in response to viral infection. Proc. Natl. Acad. Sci. U.S.A. 105, 15944–15949 (2008). 18824682 10.1073/pnas.0807707105 PMC2572935

[40] G. Schleyer., In plaque-mass spectrometry imaging of a bloom-forming alga during viral infection reveals a metabolic shift towards odd-chain fatty acid lipids. Nat. Microbiol. 4, 527–538 (2019). 30718847 10.1038/s41564-018-0336-y PMC6420086

[41] D. Schatz., Communication via extracellular vesicles enhances viral infection of a cosmopolitan alga. Nat. Microbiol. 2, 1485–1492 (2017). 28924189 10.1038/s41564-017-0024-3

[42] A. Vardi., Host-virus dynamics and subcellular controls of cell fate in a natural coccolithophore population. Proc. Natl. Acad. Sci. U.S.A. 109, 19327–19332 (2012). 23134731 10.1073/pnas.1208895109 PMC3511156

[43] X. Mari, M. E. Kerros, M. G. Weinbauer, Virus attachment to transparent exopolymeric particles along trophic gradients in the southwestern lagoon of New Caledonia. Appl. Environ. Microbiol. 73, 5245–5252 (2007). 17586679 10.1128/AEM.00762-07 PMC1950989

[44] M. J. L. Coolen, 7000 years of Emiliania huxleyi viruses in the black sea. Science 333, 451–452 (2011). 21778399 10.1126/science.1200072

[45] J. E. Lawrence, A. M. Chan, C. A. Suttle, Viruses causing lysis of the toxic bloom-forming alga Heterosigma akashiwo (Raphidophyceae) are widespread in coastal sediments of British Columbia, Canada. Limnol. Oceanogr. 47, 545–550 (2002).

[46] N. Barak-Gavish., Bacterial virulence against an oceanic bloom-forming phytoplankter is mediated by algal DMSP. Sci. Adv. 4, eaau5716 (2018). 30397652 10.1126/sciadv.aau5716 PMC6200362

[47] A. Chambouvet., Diverse molecular signatures for ribosomally ‘active’ Perkinsea in marine sediments. BMC Microbiol. 14, 110–120 (2014). 24779375 10.1186/1471-2180-14-110 PMC4044210

[48] K. M. J. Mayers., Growth and mortality of coccolithophores during spring in a temperate shelf sea (Celtic Sea, April 2015). Prog. Oceanogr. 177, 101928 (2019).

[49] R. Liu, Y. Liu, Y. Chen, Y. Zhan, Q. Zeng, Cyanobacterial viruses exhibit diurnal rhythms during infection. Proc. Natl. Acad. Sci. U.S.A. 116, 14077–14082 (2019). 31235591 10.1073/pnas.1819689116 PMC6628666

[50] F. O. Aylward., Diel cycling and long-term persistence of viruses in the ocean’s euphotic zone. Proc. Natl. Acad. Sci. U.S.A. 114, 11446–11451 (2017). 29073070 10.1073/pnas.1714821114 PMC5663388

[51] R. J. Puxty, A. D. Millard, D. J. Evans, D. J. Scanlan, Viruses inhibit CO2 fixation in the most abundant phototrophs on Earth. Curr. Biol. 26, 1585–1589 (2016). 27291056 10.1016/j.cub.2016.04.036

[52] U. Sheyn, S. Rosenwasser, S. Ben-Dor, Z. Porat, A. Vardi, Modulation of host ROS metabolism is essential for viral infection of a bloom-forming coccolithophore in the ocean. ISME J. 10, 1742–1754 (2016). 26784355 10.1038/ismej.2015.228 PMC4918435

[53] L. R. Thompson., Phage auxiliary metabolic genes and the redirection of cyanobacterial host carbon metabolism. Proc. Natl. Acad. Sci. U.S.A. 108, E757–E764 (2011). 21844365 10.1073/pnas.1102164108 PMC3182688

[54] J. Zhao, X. Zhang, Y. Hong, Y. Liu, Chloroplast in plant-virus interaction. Front. Microbiol. 7, 1565 (2016). 27757106 10.3389/fmicb.2016.01565 PMC5047884

[55] N. R. Record, D. Talmy, S. Våge, Quantifying tradeoffs for marine viruses. Front. Mar. Sci. 3, 251 (2016).

[56] F. Vincent, U. Sheyn, Z. Porat, D. Schatz, A. Vardi, Visualizing active viral infection reveals diverse cell fates in synchronized algal bloom demise. Dryad . 10.5061/dryad.h44j0zpjc. Deposited 15 February 2021. PMC7980383 33707211

[57] Luminex Corporation, IDEAS^®^ image data exploration and analysis software user's manual. https://www.luminexcorp.com/download/amnis-ideas-software-user-manual/. Accessed 8 March 2021.

[58] J. Schindelin., Fiji: An open-source platform for biological-image analysis. Nat. Methods 9, 676–682 (2012). 22743772 10.1038/nmeth.2019 PMC3855844

[59] H. Hennig., An open-source solution for advanced imaging flow cytometry data analysis using machine learning. Methods 112, 201–210 (2017). 27594698 10.1016/j.ymeth.2016.08.018 PMC5231320

[60] Y. Baran., MetaCell: Analysis of single-cell RNA-seq data using K-nn graph partitions. Genome Biol. 20, 206 (2019). 31604482 10.1186/s13059-019-1812-2 PMC6790056

[61] F. Vincent., Data from “AQUACOSM VIMS-Ehux: Core data.” Dryad . 10.5061/dryad.q573n5tfr. Accessed 5 March 2021.

[62] M. J. Frada, K. D. Bidle, I. Probert, C. de Vargas, In situ survey of life cycle phases of the coccolithophore Emiliania huxleyi (Haptophyta). Environ. Microbiol. 14, 1558–1569 (2012). 22507290 10.1111/j.1462-2920.2012.02745.x

