# Supplementary material for: Visualizing active viral infection reveals diverse cell fates in synchronized algal bloom demise
Source: Proc Natl Acad Sci U S A. 2021 Mar 11;118(11):e2021586118. doi: 10.1073/pnas.2021586118 (PMC7980383; doi:10.1073/pnas.2021586118)
Supplement: Supplementary File [file pnas.2021586118.sapp.pdf]

PNAS
www.pnas.org

Supplementary Information for

Visualizing active viral infection reveals diverse cell fates in synchronized algal bloom demise.

Flora Vincent, Uri Sheyn, Ziv Porat, Daniella Schatz, Assaf Vardi*

*Corresponding author: Assaf Vardi
Email: assaf.vardi@weizmann.ac.il

This PDF file includes:

Figures S1 to S14
Table S1
Dataset caption for Probe_Sequences.xlsx
SI References

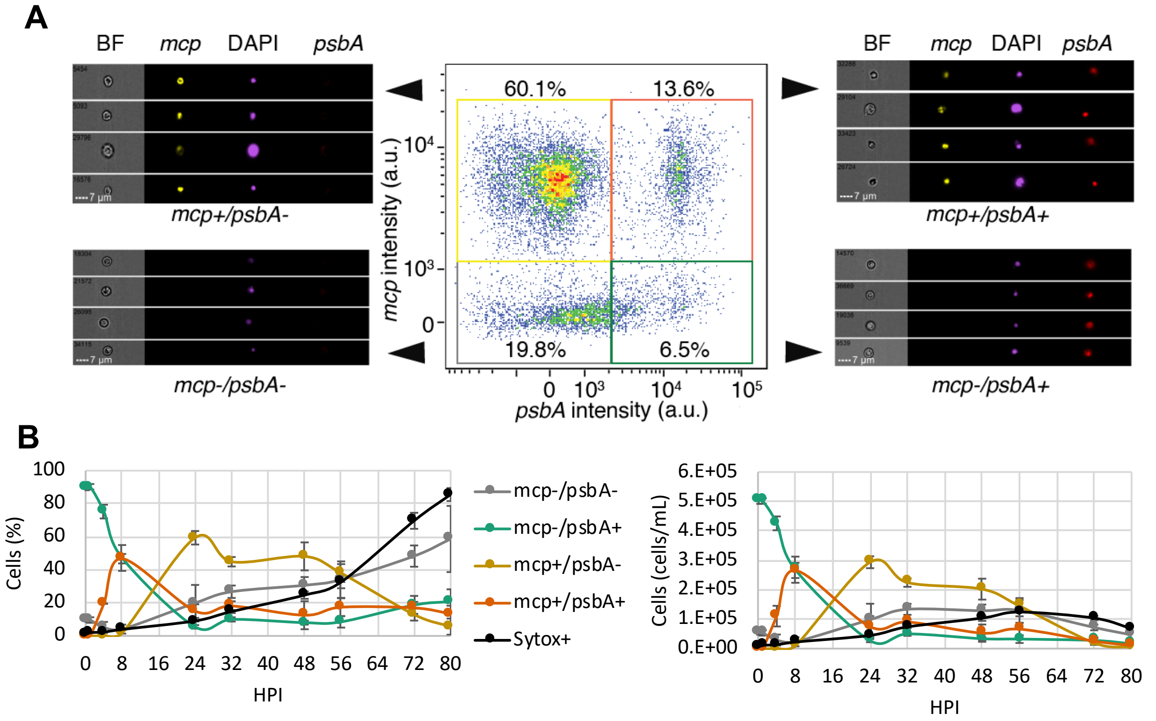

Fig. S1. Dynamics of subpopulations during infection in EhV201. (A) The central panel represents co-expression of host *psbA* and viral *mcp* (X and Y axes, respectively in arbitrary units of fluorescence) in an infected *E. huxleyi* culture 24 hpi. Four subpopulations, representing distinct transcriptional states, are observed: *mcp*-/*psbA*+ (green gate), *mcp*+/*psbA*+ (red gate), *mcp*+/*psbA*- (yellow gate) and *mcp*-/*psbA*- (grey gate). Single-cell morphology and fluorescent signal of each subpopulation is presented, showing brightfield image (BF), *mcp* (yellow), DAPI (purple) and *psbA* (red). (B) Proportion of cells in each gate through time (left panel) and absolute abundance in cell/mL of cells in each gate (right panel), including Sytox+ dynamics.

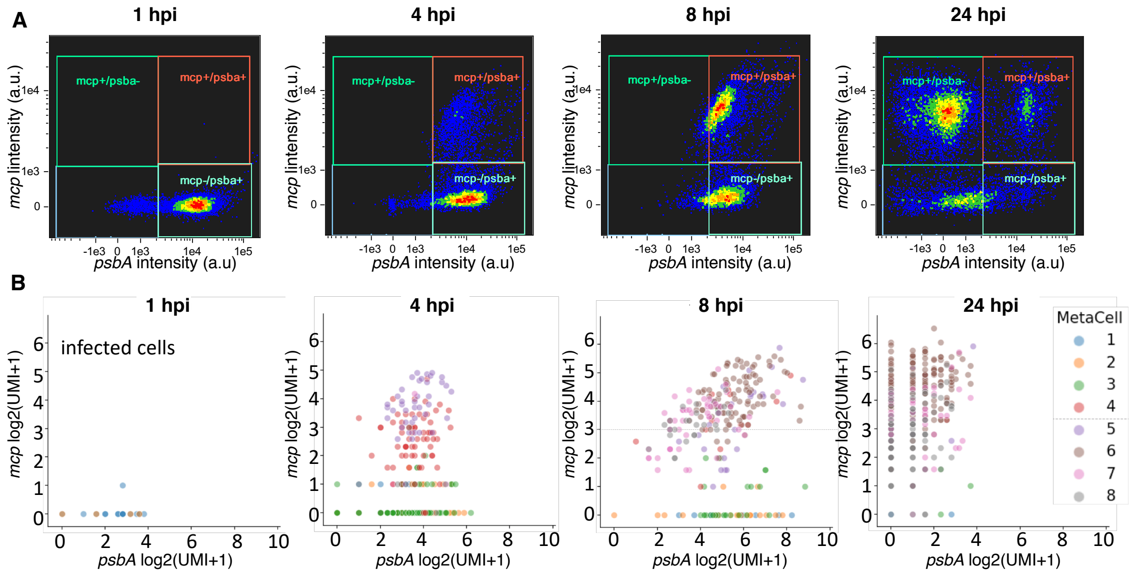

Fig. S2. Comparison of smFISH with single-cell RNA-Seq. (A) Time course of transcriptional states in EhV201 based on imaging flow cytometry and smFISH with *psbA* (x axis) and *mcp* (y axis) probes. (B) Time course of single cell RNA-Seq for the same time points of an EhV201 infection (1). Each dot is an infected cell and is plotted with respect to the number of unique *mcp* and *psbA* mRNA counts (Unique Molecular Identifier, or UMI). Each cell is colored by the metacell it belongs to, where a metacell can be defined as a group of infected cells that form a cohesive group based on their total individual RNA expression. Metacell 1 (blue) represents cells in early stage of the viral program, and metacell 8 (grey) represents cells in the last stage of the viral program. For example, at 4 hpi, most of the cells in the *mcp*-/*psbA*+ gate belong to metacells 1-3, whereas cells in the *mcp*+/*psbA*+ gate correspond to cells belonging to metacells 4-7.

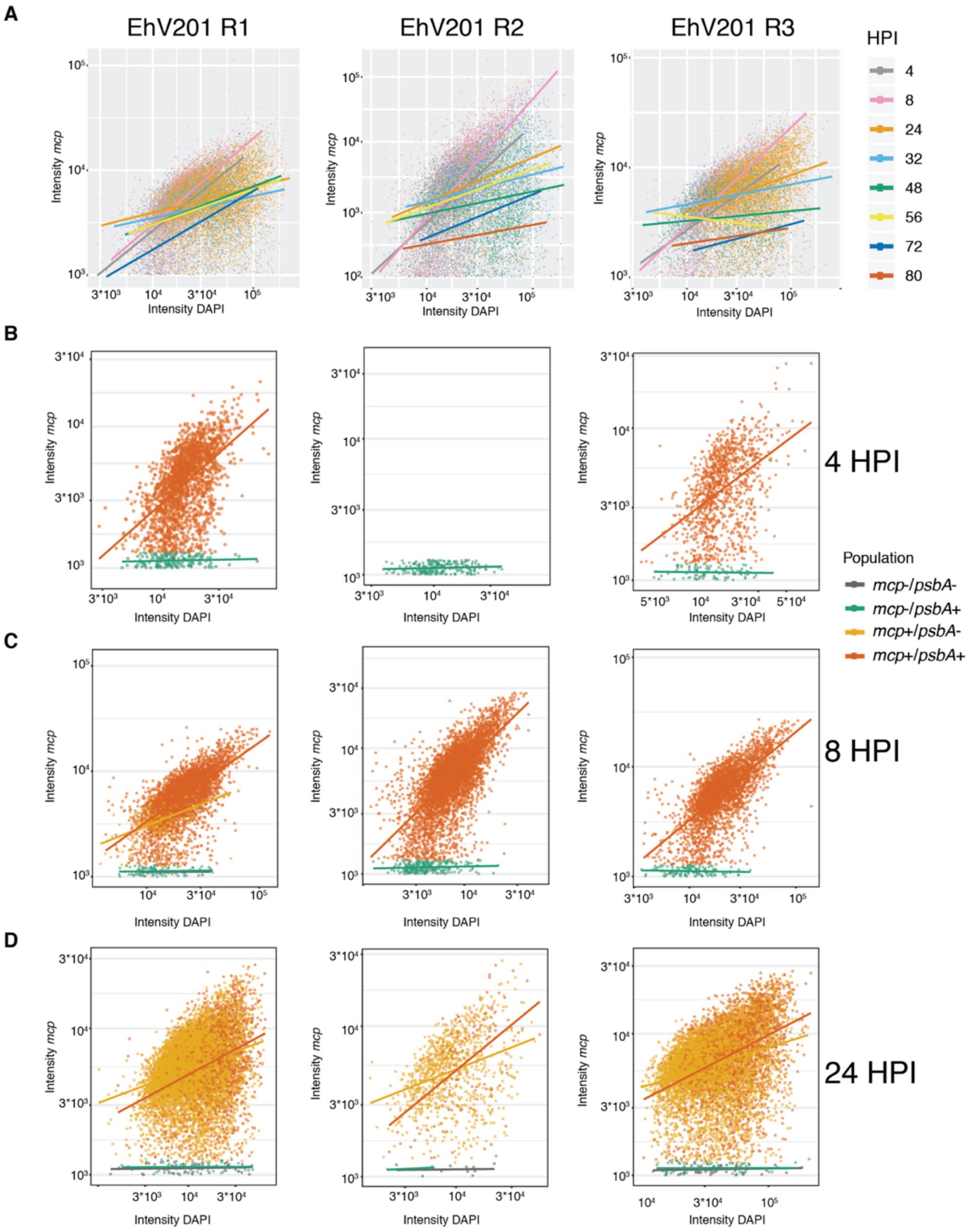

Fig. S3. *mcp* and DAPI intensities in single cells throughout infection. (A) DAPI intensity versus *mcp* intensity of single cells in EhV201 Replicate 1,2,3. Colors represent different timepoints. Slopes were plotted based on the smoothed “lm” function (geom_smooth() in R). (B) DAPI versus *mcp* for EhV201 Replicate 1,2,3 at 4 hpi colored per transcriptional subpopulation. (C) DAPI versus *mcp* for EhV201 Replicate 1,2,3 at 8 hpi colored per transcriptional subpopulation. For cells within the *mcp*+/*psbA*+ population, fluorescent intensities of the *mcp* and DAPI staining were fitted using the "lm()" function in R to fit linear models. For the three replicates, the slope value was 0.2 with a

p-value $< 2e-16$ (D) DAPI versus *mcp* for EhV201 Replicate 1,2,3 at 24 hpi colored per transcriptional subpopulation.

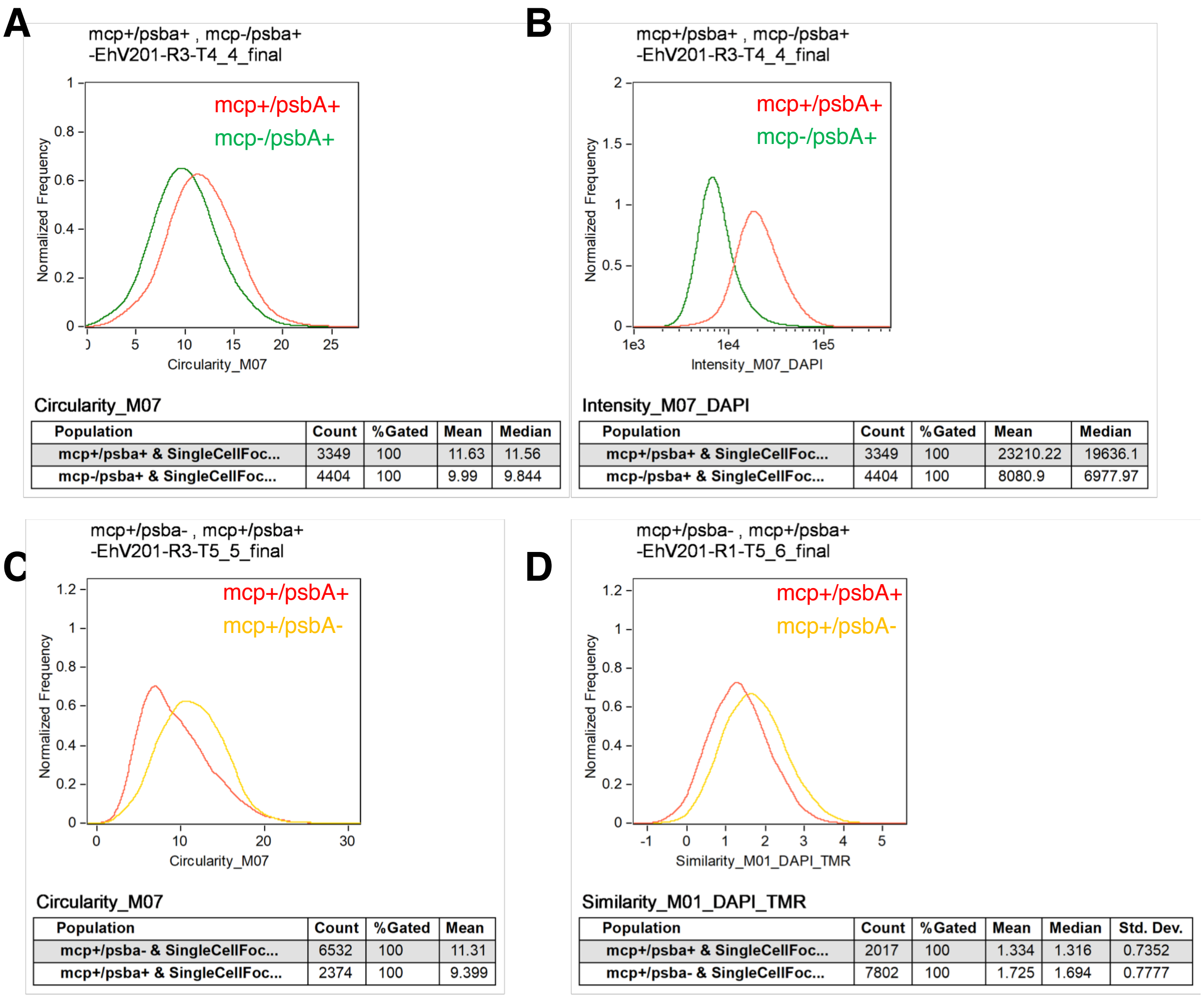

Population	Count	%Gated	Mean	Median
mcp+/psba+ & SingleCellFoc...	3349	100	11.63	11.56
mcp-/psba+ & SingleCellFoc...	4404	100	9.99	9.844

Population	Count	%Gated	Mean	Median
mcp+/psba+ & SingleCellFoc...	3349	100	23210.22	19636.1
mcp-/psba+ & SingleCellFoc...	4404	100	8080.9	6977.97

Population	Count	%Gated	Mean
mcp+/psba- & SingleCellFoc...	6532	100	11.31
mcp+/psba+ & SingleCellFoc...	2374	100	9.399

Population	Count	%Gated	Mean	Median	Std. Dev.
mcp+/psba+ & SingleCellFoc...	2017	100	1.334	1.316	0.7352
mcp+/psba- & SingleCellFoc...	7802	100	1.725	1.694	0.7777

Fig. S4. Morphology of photosynthetically active infected and non-infected cells. (A, B) Analysis of DAPI circularity and intensity respectively in *mcp*+/*psbA*+ cells (red) and *mcp*-/*psbA*+ (green) 8 hpi in biological replicate 3. (C, D). Analysis of DAPI circularity and colocalization between DAPI and *mcp* signals respectively in *mcp*+/*psbA*- cells (yellow) and *mcp*+/*psbA*+ (orange) 24 hpi in biological replicate 3.

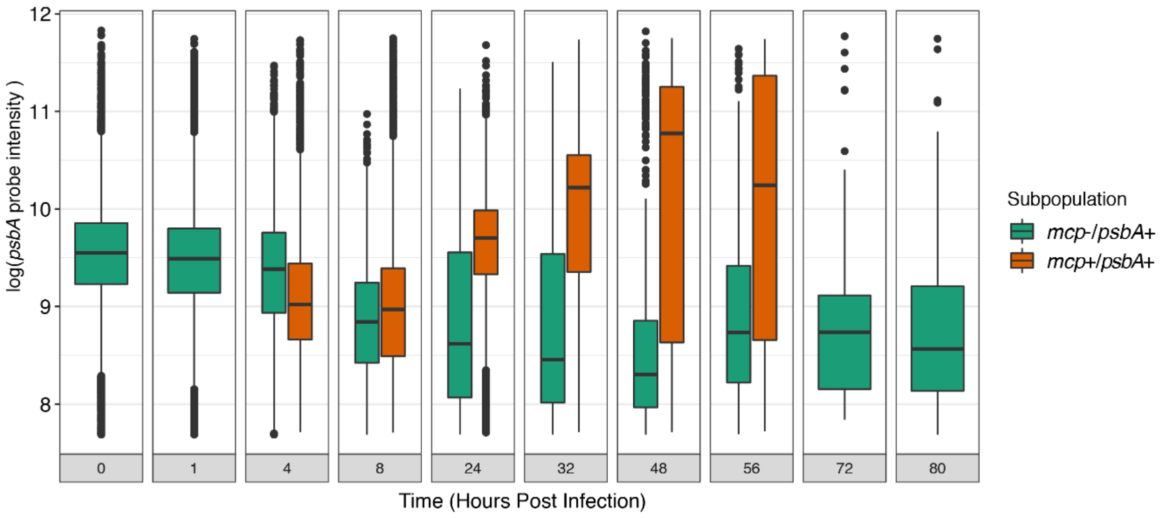

Fig. S5. Temporal dynamics of *psbA* intensities in *psbA*+ subpopulations of an infected culture, showing box plots of *psbA* probe intensities in *mcp*+/*psbA*+ (red) and *mcp*-/*psbA*+ (green) sub populations over time of infection (hpi). *psbA* intensity was significantly different (p-value < 10^{-8}) in the infected subpopulation at all times points where infected cells are present, and was tested in R with pairwise comparisons using Wilcoxon rank sum test and Benjamini Hochberg adjusted p-values.

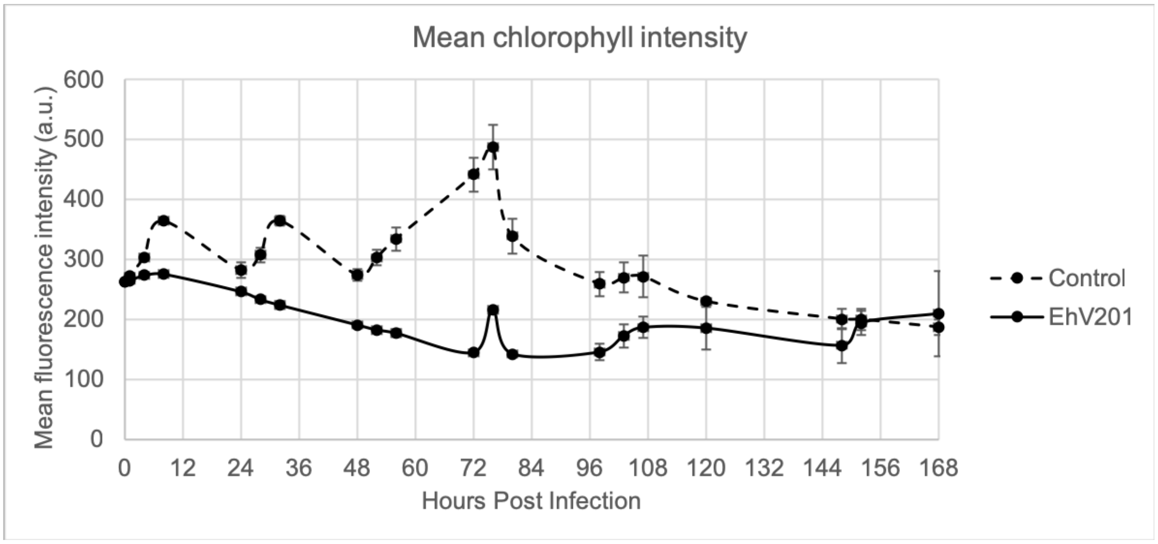

Fig. S6. Chlorophyll mean intensity during infection. Cell counts are based on chlorophyll fluorescence detected by flow cytometry. The cell gate selects cells that have high chlorophyll intensity (> $10^{\wedge 4}$ a.u). However, the mean chlorophyll intensity of that population can fluctuate as shown above between non-infected (dashed line), and infected (full line) cultures. Cells were infected at 9 a.m (0 hpi). Mean chlorophyll intensity was significantly different between infected and non-infected cultures. T-tests use Satterthwaite's method ($P < 0.001$).

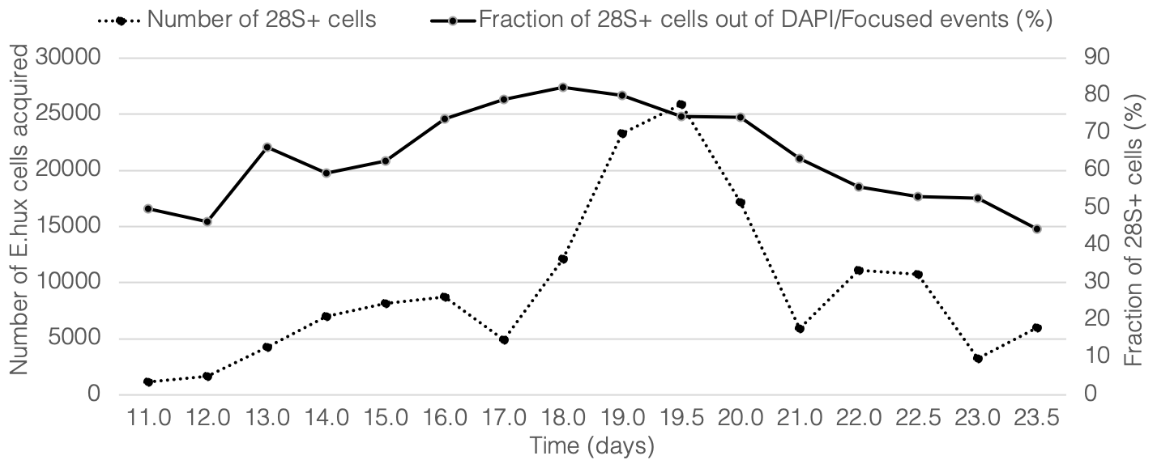

Fig. S7. Adapting smFISH to specifically target *E. huxleyi* in environmental samples.

By designing a fluorescent probe that specifically targets the 28S ribosomal region of *E. huxleyi*, we were able to identify *E. huxleyi* cells in samples originating from a natural complex community. Out of all DAPI+ events (cells with intensity value in the DAPI channel higher than $1.1*10^4$ a.u), the absolute number of *E. huxleyi* cells (left axis, dotted line) analyzed at each time point after day 16 was above 5000 cells, reaching 25000 single cells on day 19.5 (evening sample). The fraction of *E. huxleyi* cells (defined as 28S+ based on rRNA fluorescence, right axis, straight line) ranged between 40% at the beginning and end of the bloom to above 80% at the peak of the *E. huxleyi* bloom between days 17 and 19. DAPI+ events include all alga/grazers containing dsDNA that are below 40 micron in size.

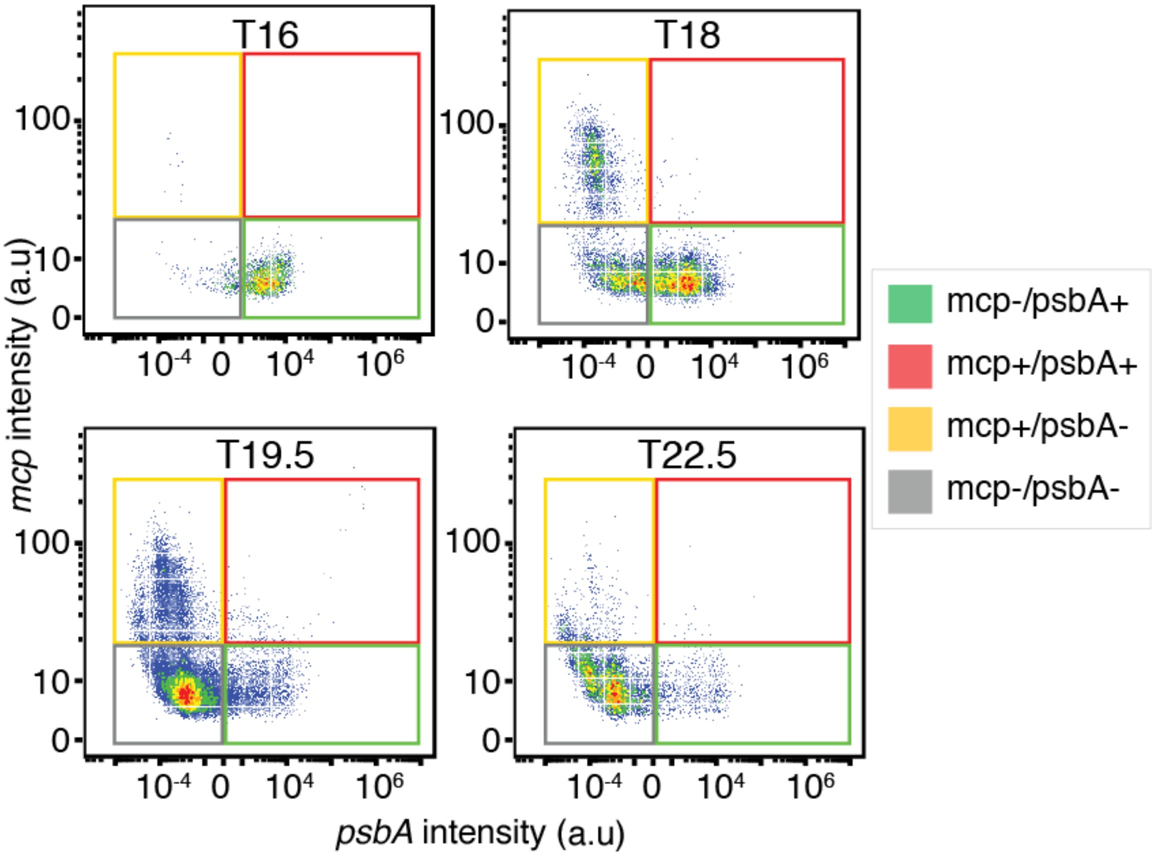

Fig. S8. Defining *mcp*/*psbA* subpopulations in environmental samples. To investigate *E. huxleyi* virocell heterogeneity during bloom succession, we plotted host and virus mRNA expression in parallel at different time points post infection (T19.5 and T22.5 represent evening samples). The X axis represents the value of the probe intensity (in fluorescent arbitrary units) targeting the *psbA* host gene, and the Y axis represents the value of the probe intensity targeting the viral *mcp* gene. Using a threshold of 20 arbitrary units of max pixel in the *mcp* channel to define *mcp*+, cells and 0 arbitrary units of fluorescence intensity in the *psbA* channel to define *psbA*+ cells, we define four subpopulations as a combination of *mcp* and *psbA* signals.

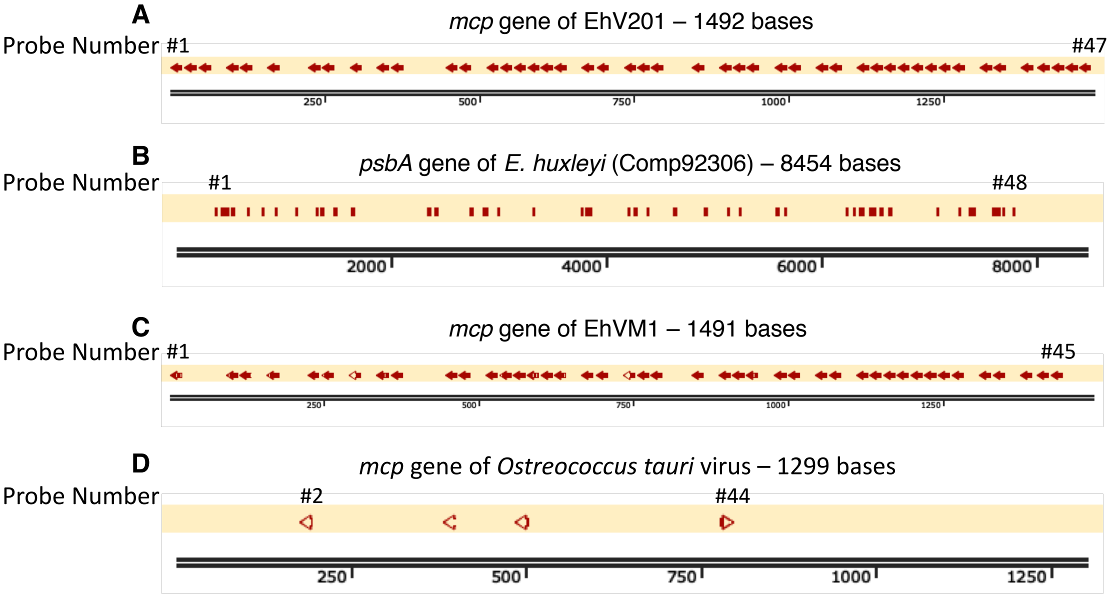

Fig. S9. Mapping of each smFISH probe against the reference host and viral genes. The smFISH technique identifies a single mRNA based on the binding of multiple small probes targeted to different locations of the mRNA of interest. A minimum of 20 probes of 20 nucleotides each are coupled to fluorophores. All the fluorescent probes of a given gene are used as a mixture each time we target the expression of that gene, to amplify the signal. (A) Mapping of each *mcp* probe (in red) against the full *mcp* sequence of EhV201, the virus that was used for infection in the lab experiments (in black). Probe #1 is located at the beginning of the sequence and Probe #47 is the last probe mapped to the sequence. The 47 probes mapped within the sequence and all had a 100% match within the EhV201 sequence. (B) Mapping of each *psbA* probe (in red) against the full *psbA* sequence of *E. huxleyi*. Probe #1 is located at the beginning of the sequence and Probe #48 is the last probe mapped to the sequence. The 48 probes mapped within the sequence and all had a 100% match within the *psbA* sequence. Exact positions of the probes are given in Probe _Sequences.xlsx. (C) Mapping of each *mcp* probe (in red) against the full *mcp* sequence of the most abundant virus found in the mesocosm, EhVM1 (in black). Probe #1 is located at the beginning of the sequence and Probe #45 is the last probe mapped to the sequence. Out of 47 probes, 32 probes had a 100% match over 20 nucleotides to EhVM1. Probes #9 and #22 had 100% match over 19 and 18 nucleotides respectively. Seven probes had one mismatch (Probes #4, #6, #8, #10, #15, #18, #19) and two probes had two mismatches (Probes #1, #17) within the 20 nucleotides. Those mismatches within the probe sequence will marginally decrease the signal, however fluorescence is high enough to be detected in the microscope, flow cytometry and the Imagestream. Probes #2, #3, #46, #47 did not align. (D) Mapping of the 47 *mcp* probes against the *mcp* gene of *Ostreococcus tauri* virus. Arrows indicate the direction of the mapping. Filled arrows indicate areas

of identity, while empty arrows indicates areas of mismatch. Only four probes were mapped, with less than 17 nucleotides identity.

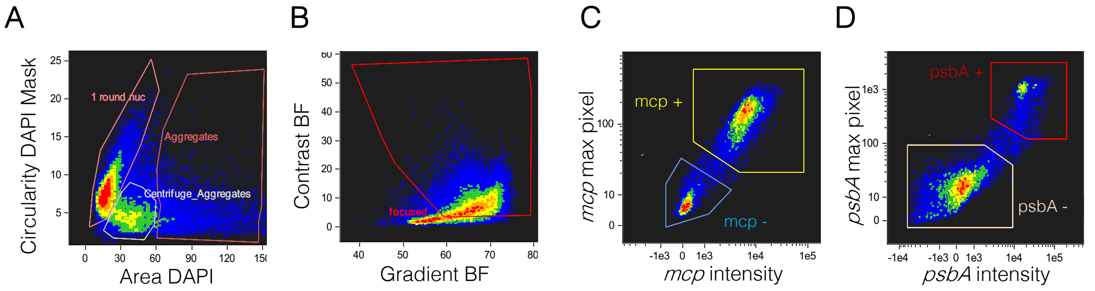

Fig. S10. Pipeline of single cell identification in multispectral imaging flow-cytometer. (A) Identification of single cells based on the area of the DAPI in each event versus the circularity of the DAPI mask. (B) Identification of focused single cells based on the gradient in the brightfield versus the contrast. Both gradient and contrast measure the sharpness quality of an image by detecting large changes of pixel values in the image (C, D). Within the focused single cells, positively stained populations of *mcp* and *psbA*, respectively, can be defined by the intensity (sum of all pixel intensities in a single cell) versus the max pixel (value of the highest pixel) of each event.

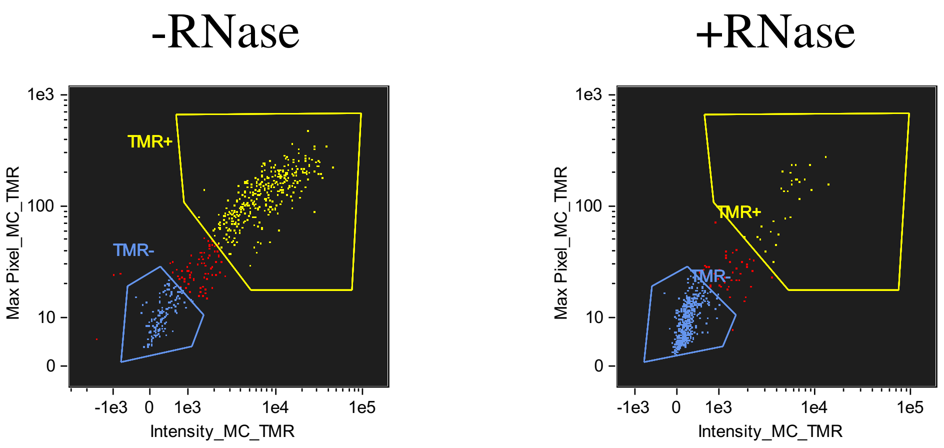

Intensity_MC_TMR, Max Pixel_MC_TMR

Population	Count	%Gated
focused & DAPI+ & 1 nuc	559	100
TMR- & focused & DAPI+ & 1 nuc	139	24.9
TMR+ & focused & DAPI+ & 1 nuc	344	61.5

Intensity_MC_TMR, Max Pixel_MC_TMR

Population	Count	%Gated
focused & DAPI+ & 1 nuc	594	100
TMR- & focused & DAPI+ & 1 nuc	517	87
TMR+ & focused & DAPI+ & 1 nuc	32	5.39

Fig. S11. Validation that the smFISH signal derived mainly from mRNA transcript level. TMR is the abbreviation for "Tetramethylrhodamine" and is the fluorophore used to visualize the *mcp* transcripts. The non-treated sample shows 61.5% of *mcp*+ cells, whilst the RNase treated sample showed less than 5% of *mcp*+ cells that could be noise considering the number of cells. This suggests that our smFISH probe catches mainly RNA and not DNA.

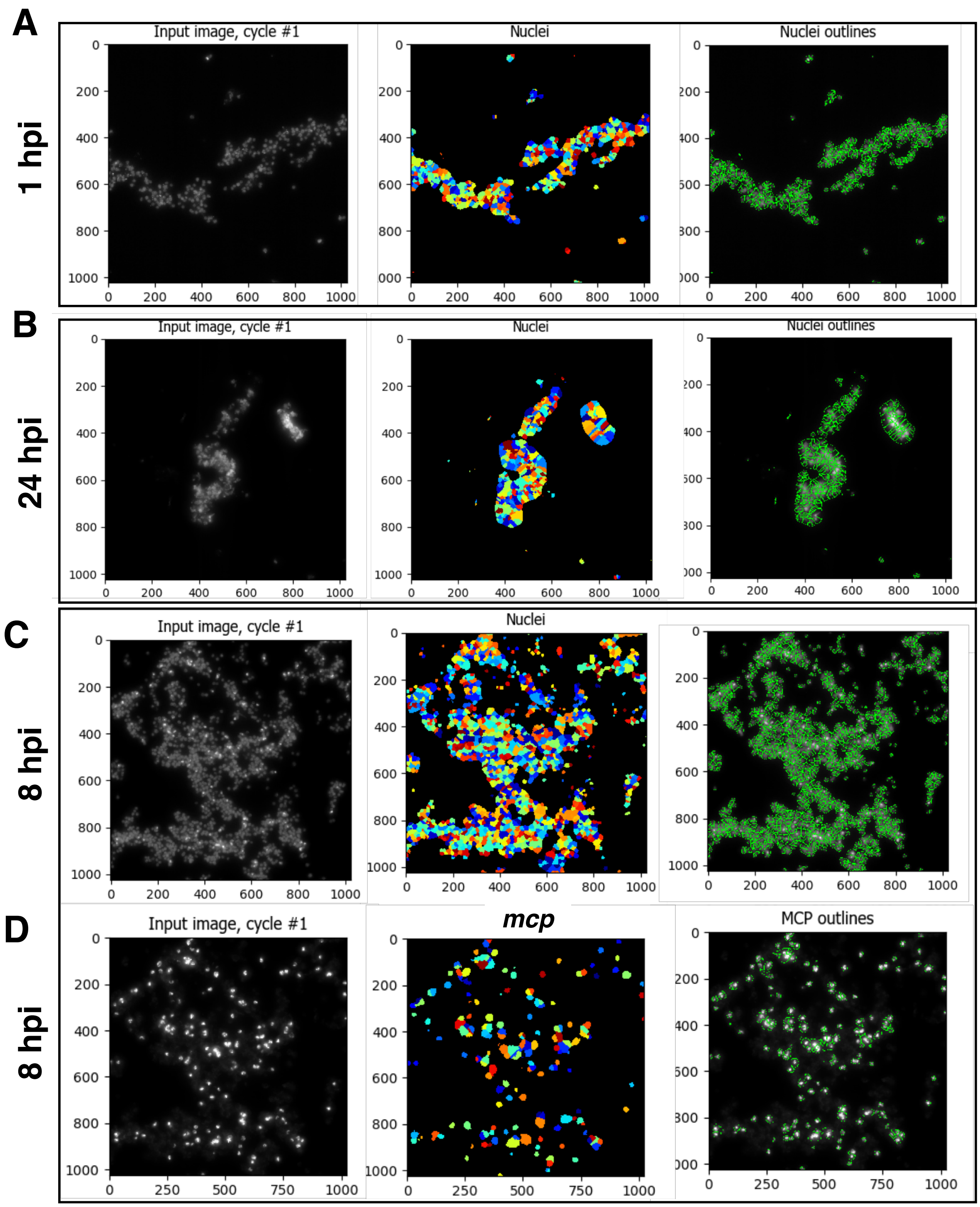

Fig. S12. smFISH analysis of infected cells in microscopy data using CellProfiler. CellProfiler (2) was used to analyze microscopy data. In particular, the "PercentPositive" pipeline was applied. For each sample, several channels are collected including DAPI, TMR (*mcp* gene), Cy5 (*psbA* gene), and FITC (DioC6 membrane stain). (A, B) DAPI channel images of infected *E. huxleyi* cells at 1

and 24 hpi. CellProfiler identifies nuclei based on the DAPI signal, performs nuclei segmentation and then draws nuclei outline in green. (C, D) DAPI and *mcp* channel images respectively of infected *E. huxleyi* cells at 8 hpi. Here, we show how the *mcp* images are then used to count how many cells are positively expressing the *mcp* gene. A ratio of positive events, defined by the number of cell displaying *mcp* signal over total number of nuclei, is given as final output.

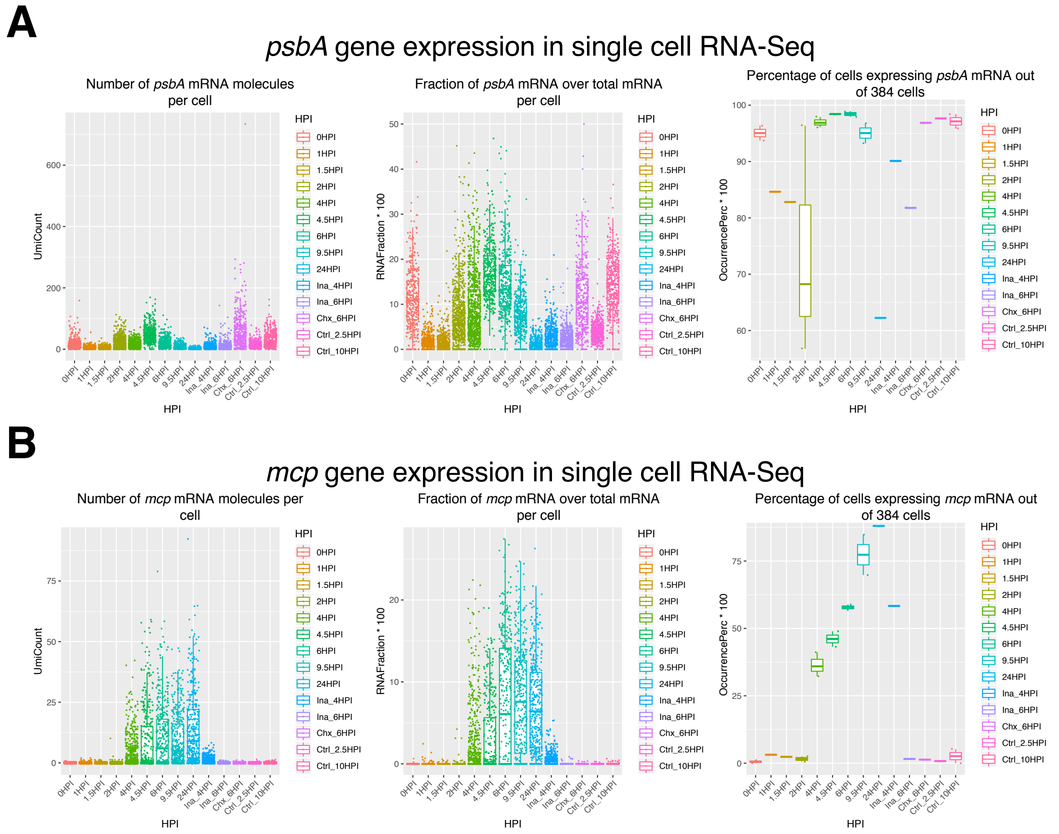

Fig. S13. Gene expression of *psbA* and *mcp* in single cell RNA-seq sequencing data. Expression of *psbA* and *mcp* in single cell RNA-seq dataset during viral infection for a time course between 0-24 hpi for (A) *psbA* (B) *mcp*. This includes control (non-infected cultures), UV inactivated viruses, and cycloheximide (translation inhibitor) experiments reported in (1). From left to right: absolute unique molecular identifier (UMI) count per cell; ratio between gene UMI count and total UMI count in each cell; fraction of cells that show UMI for that gene. Data extracted from (1).

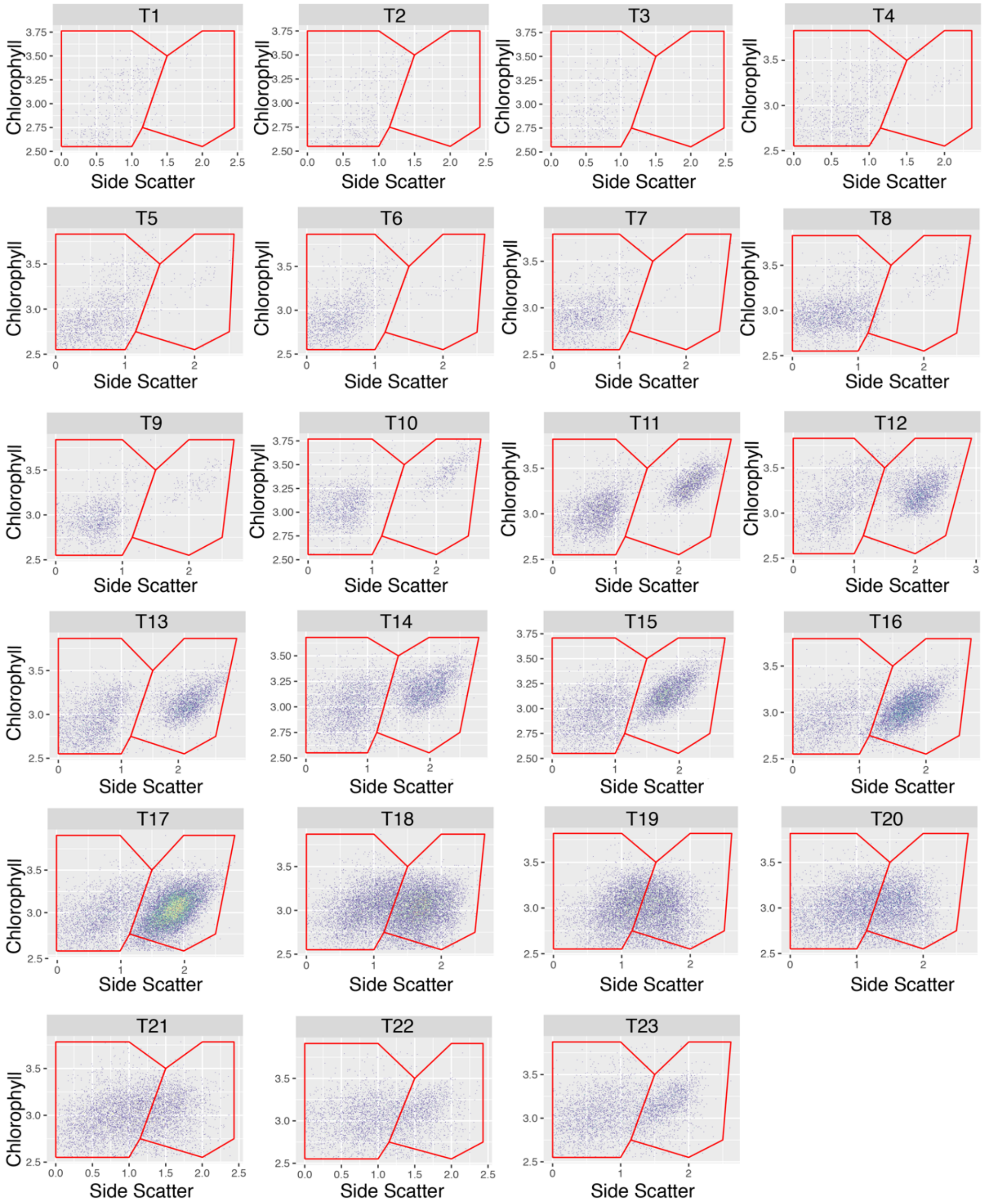

Fig. S14. Flow cytometry gates used for detection of *E. huxleyi* cells in the mesocosm experiment. Flow cytometry gates used for counting calcified (right gate) and non-calcified (left gate) nano-eukaryotes based on side scatter versus chlorophyll intensity shown here between day 1 and 23 which correspond to the bloom and demise phase of Bag 4. Absolute abundance of calcified *E. huxleyi* is presented in cells mL^{-1} and was obtained by normalizing to the sampled volume for each acquisition.

MPN (infectious particles mL^{-1})	7.6E+07
E. huxleyi concentration (cells mL^{-1})	557000
Volume of virus used (mL)	38
MOI	6.4

Table S1. Calculation of virus:host ratio using the MPN method. The concentration of infectious particles was calculated based on MPN method (3), performed on day 0 of the time course of infection, with the same cells and viral stock used for the experiment in Fig. 1D-E. The final virus:host ratio performed in the experiment was of 6.4.

Dataset caption for Probe_Sequences.xlsx: This file contains all the necessary data regarding the sequences and mapping of smFISH probes of the two mRNAs targeted of this study. **Tab "psbA"**: This tab contains the name (Column A) and sequence (Column B) of the 48 individual probes that have been used to target the *psbA* host gene, including their position in the reference sequence (Column C). The reference sequence of *psbA* used to design the probes is given in Column E. **Tab "mcp":** This tab contains the name (Column A) and sequence (Column B) of the 47 individual probes that have been used to target the *mcp* host gene, including their position in the reference sequence (Column C). The reference sequence of *m*cp used to design the probes is given in Column E.

SI References

1. C. Ku, *et al.*, A single-cell view on alga-virus interactions reveals sequential transcriptional programs and infection states. *Sci. Adv.* **6**, eaba4137 (2020).
2. H. Hennig, *et al.*, An open-source solution for advanced imaging flow cytometry data analysis using machine learning. *Methods* **112**, 201–210 (2017).
3. B. Jarvis, C. Wilrich, P. T. Wilrich, Reconsideration of the derivation of Most Probable Numbers, their standard deviations, confidence bounds and rarity values. *J. Appl. Microbiol.* **109**, 1660–1667 (2010).
